# The Influence of Printing Orientation on the Properties of 3D-Printed Polymeric Provisional Dental Restorations: A Systematic Review and Meta-Analysis

**DOI:** 10.3390/jfb16080278

**Published:** 2025-07-31

**Authors:** Firas K. Alqarawi

**Affiliations:** Department of Substitutive Dental Sciences, College of Dentistry, Imam Abdulrahman Bin Faisal University, Dammam 31441, Saudi Arabia; fkalqarawi@iau.edu.sa

**Keywords:** provisional dental restorations, 3D printing, CAD/CAM, fracture strength, Printing orientation, wear resistance, printing angulation, mechanical properties, physical properties, surface roughness, provisional fixed dental prosthesis, flexural strength, color stability, water absorption, modulus of elasticity, hardness

## Abstract

Three-dimensional printing is commonly used to fabricate provisional dental restorations. Studies have reported that changes in printing orientation affect the physical and mechanical properties of 3D-printed polymeric provisional restorations; however the findings have been inconsistent. Therefore, this systematic review and meta-analysis aims to analyze the articles evaluating the influence of printing orientation on the physical and mechanical properties of 3D-printed polymeric provisional dental restorations. Recommendations provided by the Preferred Reporting Items for Systematic Reviews and Meta-Analyses (PRISMA) guidelines were followed to structure and compose the review. The PICO (Participant, Intervention, Comparison, Outcome) question ordered was: ‘Do 3D-printed provisional dental restorations (P) printed at various orientations (except 0°) (I) exhibit similar physical and mechanical properties (O) when compared to those printed at a 0° orientation (C)?’. An electronic search was conducted on 28 and 29 April 2025, by two independent researchers across four databases (MEDLINE/PubMed, Scopus, Cochrane Library, and Web of Science) to systematically collect relevant articles published up to March 2025. After removing duplicate articles and applying predefined inclusion and exclusion criteria, twenty-one articles were incorporated into this review. Self-designed Performa’s were used to tabulate all relevant information. For the quality analysis, the modified CONSORT scale was utilized. The quantitative analysis was performed on only fifteen out of twenty-one articles. It can be concluded that the printing orientation affects some of the tested properties, which include fracture strength (significantly higher for specimens printed at 0° when compared to 90°), wear resistance (significantly higher for specimens printed at 90° when compared to 0°), microhardness (significantly higher for specimens printed at 90°and 45° when compared to 0°), color stability (high at 0°), and surface roughness (significantly higher for specimens printed at 45° and 90° when compared to 0°). There were varied outcomes in terms of flexural strength and elastic modulus.

## 1. Introduction

Provisional restorations play a crucial role in fixed dental prosthesis treatment by protecting pulp, maintaining periodontal tissue, and providing function and esthetics [1,2]. The materials used should possess the necessary physical and mechanical properties to prevent failures [3,4]. Technological advancements in the dental field have helped dentists in various stages of treatment, including treatment planning, tooth preparation, and the fabrication of provisional and definitive prostheses. These advancements have improved overall efficiency and enabled the delivery of high-quality treatment to patients [5,6,7,8].

Computer-aided design and computer-aided manufacturing (CAD/CAM) are commonly used in the fabrication of indirect provisional and definitive restorations [9]. Compared to milling, the 3D printing technique involves less material wastage, no wear of milling tools, and facilitates the faster fabrication of the prosthesis. Additionally, 3D printing machines that are lower in cost help reduce the overall cost of the prosthesis [10,11,12].

In 3D printing the photo polymerization of the printed materials in dentistry can be conducted by various methods, which involve stereolithography (SLA) technology-based printers (using a UV laser to photo polymerize), digital light processing (DLP) technology-based printers (using a digital projector screen), and printers using a liquid-crystal display (LCD) as the light source [13,14]. Three-dimensional printing is frequently used to manufacture provisional fixed dental prostheses, maxillofacial prostheses, removable dental prostheses, and definitive fixed dental prostheses [11,15,16,17,18,19]. Studies have reported that 3D-printed provisional dental prostheses have good physical properties, mechanical properties, internal fit, and marginal fit when compared to conventionally fabricated and milled prostheses [20,21].

Three-dimensional printers come with a set of printing parameters that can be altered. Generally, the manufacturer recommends setting these parameters based on the type of 3D-printed resin used. Some of the parameters that the operator can adjust in a 3D printer include printing layer thickness, printing angulation, and the position of an object on the printing platform [22,23,24].

The previously published literature has reported that changing printing factors, such as layer thickness and orientation, can alter the properties of the prosthesis, reduce orienting time, and allow for better positioning, thereby increasing the number of objects on the platform [25,26,27,28].

Anisotropy is the phenomenon where the physical properties of materials differ according to the printing orientation [28]. Various studies have been conducted to assess the influence of printing orientation on the physical and mechanical properties of various dental prosthetic devices, including dental aligners, occlusal devices, removable dental prostheses, fixed dental prostheses, dental ceramics, surgical guides, and denture teeth [25,26,27,28,29,30,31,32]. Some studies have reported a significant effect on the physical and mechanical properties of 3D-printed provisional resins [33,34,35,36,37,38,39], whereas others have reported minimal or no effect [40,41]. Alharabi et al. [34] and Farkas et al. [35] reported that the compressive strength of provisional resins is influenced by printing orientation, with compressive strength higher for specimens printed at 90° compared to those printed at 0°. Contrasting results were reported when fracture resistance was evaluated. Alkhateeb et al. [42] reported the highest fracture resistance for specimens printed at 45°, whereas Aljehani et al. [36] reported the highest fracture resistance for specimens printed at 90°. A study by Queiroz et al. [43] reported the highest microhardness for specimens printed at 45°, followed by those printed at 90° and 0°. Similar results were reported by de Castro et al. [25] for Nanolab 3D resin and Mudhaffer et al. [39] for Nextdent CB MFH resin. For the Cosmos Temp-DLP [25], Dima CB temp [39], and GC temp print [39] resins, the specimens printed at 90° displayed higher microhardnesses, followed by specimens printed at 45 ° and 0°. Lee et al. [33] and Espinar et al. [44] reported that a color change is influenced by print orientation. Lee et al. reported the highest color change with specimens printed at 90°, followed by those printed at 45° and 0°. Contrastingly, the study by de Castro et al. [40] reported that printing orientation has no influence on the color change. Studies by Queiroz et al. [43], de Castro et al. [25], and Mudhaffer et al. [39] reported the effects of printing orientation on microhardness, which also varies according to the type of printing material. Lee et al. [45] and Wan et al. [46] reported contrasting results when they compared the wear volume loss (wear resistance) of the tested 3D-printed specimens. Various studies [25,38,43,47,48,49,50] have reported that flexural strength is also affected by printing orientation, which varies according to the tested materials. However, a study by Espinar et al. [41] reported no influence of printing orientation on the flexural strength of 3D-printed provisional resins. Studies by Nasiry Khanlar et al. [37], de Castro et al. [40], Ortega et al. [51], and de Gois Moreira et al. [49] reported a higher surface roughness for tested materials when printed at 45° compared to those printed at 90° and 0°. However, Queiroz et al. [43] reported the highest surface roughness for specimens printed at 0°. Similarly, contrasting results were reported for microhardness [25,39,43], elastic modulus [41], and tensile strength [35]. The results of these studies are conflicting. Therefore, it is crucial to assess this parameter. There is no known meta-analysis that evaluates the influence of printing orientation on the physical and mechanical properties of 3D-printed provisional dental restorations. The results of this systematic review can guide the selection of the best printing orientation when fabricating 3D-printed provisional dental restorations. The tested null hypothesis is that there is no effect of changing printing orientation on the physical and mechanical properties of 3D-printed provisional polymeric dental restorations.

## 2. Materials and Methods

The review protocol was registered with the International Prospective Register of Systematic Reviews (Prospero registration no: CRD420251041876). The review was configured and organized as per the recommendations provided by the Preferred Reporting Items for Systematic Reviews and Meta-Analyses (PRISMA) [52].

### 2.1. Article Selection

The article selection criteria are itemized in Table 1.

### 2.2. Exposure and Outcome

The exposure in this study was the 3D-printed provisional dental restorations printed at various printing orientations. The outcome was the assessment of their physical and mechanical properties. The focused PICO (Participant, Intervention, Comparison, Outcome) question was: ‘Do 3D-printed provisional dental restorations (P) printed at various orientations (except 0°) (I) exhibit similar physical and mechanical properties (O) when compared to those printed at a 0° orientation (C)?’

### 2.3. Information Sources, Search Strategy, and Data Extraction

**Figure 1:**
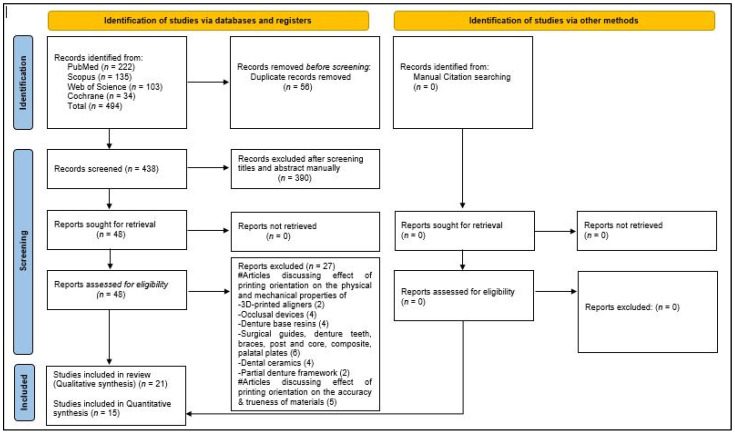
PRISMA flow-chart representing the article selection strategy.

An electronic search was conducted by two independent researchers (F.A. and M.A.A.) across four databases (MEDLINE/PubMed, Scopus, Cochrane Library, and Web of Science) to collect the relevant articles systematically. The search strings used based on the PICO questions were: ‘3D-printed provisional restorations’ AND ‘printing orientations’ AND ‘physical and mechanical properties.’ The article search was conducted on April 28 and 29, 2025, using truncation and Boolean operators. Minor adjustments were applied to the search strings to comply with the requirements of each database (Supplementary Table S1). The search was limited to articles published in the English language up to March 2025. The two researchers removed the duplicate articles and went through the titles and abstracts of the remaining articles to select the appropriate studies. Later, these researchers searched the gray literature and manually reviewed bibliographies and other relevant articles to ensure that no pertinent articles were overlooked. The full texts of the shortlisted studies were reviewed by each researcher individually to select the relevant articles (Figure 1). Any differences between the two researchers related to article selection were discussed and resolved after consultation with a third researcher (H.A.A.). F.A. created data extraction charts to collect all the relevant information (Table 2). Individual tables were used to compile detailed data for each physical and mechanical property tested (Table 3, Table 4, Table 5, Table 6, Table 7, Table 8, Table 9, Table 10, Table 11, Table 12 and Table 13). The information includes the details of the outcomes for the tested property under different printing orientations, the testing machines, the conclusions, and the authors’ suggestions. (Table 3, Table 4, Table 5, Table 6, Table 7, Table 8, Table 9, Table 10, Table 11, Table 12 and Table 13). The extracted data were reviewed and validated by the two researchers (M.A.A. and H.A.A.) to ensure that no relevant information was missed.

### 2.4. Quality Assessment of Included Studies

A modified CONSORT scale for in vitro studies [54,55] was used to assess the quality of the selected studies. The scale includes fourteen items under different sections of the manuscript (Abstract, Introduction, Methods, Results, Discussion, and Other Information) [54,55] (Supplementary Table S2).

### 2.5. Quantitative Assessment

Review Manager 5.4.1 was used to perform the quantitative analysis in Non-Cochrane Review mode [56]. The different physical and mechanical properties of provisional restorations printed at different angulations were compared. An inverse variance was used to calculate the mean difference using a random effects model. The mean difference was used because all measurements were in the metric system and uniform. Since the studies were performed in different settings, the random effects model was used. A 95% confidence interval was used to express the results of individual studies and the pooled result. The heterogeneity was measured using the Chi-squared test, with a *p*-value < 0.05 being considered significant. I2 was also calculated and reported in the results. Separate forest plots were used to compare 0° with 45° and 90°, respectively.

## 3. Results

### 3.1. Identification and Screening

Four hundred ninety-four hits were found after a preliminary electronic search of the four databases (PubMed: 222; Scopus: 135; Web of Science: 103; Cochrane: 34). Of these, 56 titles were duplicates and were subsequently removed. The remaining articles were reviewed, and it was determined that 390 articles did not meet the selection criteria and were thus rejected. The full texts of the remaining 48 articles were reviewed. A manual search of the references of these articles was conducted to identify any additional articles, but none were found. In total, 27 articles were excluded, and of these 22 articles discussed the effect of printing orientation on the physical and mechanical properties of various other materials and devices (3D-printed aligners [29,57], occlusal devices [30,58,59,60], denture base resins [31,61,62,63], surgical guides [64], denture teeth [65], braces [66], post and core [67], composite [68], palatal plates [32], dental ceramics [28,69,70,71], and partial denture frameworks [62,72]. Five articles discussed the influence of printing orientation on the accuracy of the materials [73,74,75,76,77]. Finally, 21 articles were selected for qualitative analysis [25,33,34,35,36,37,38,39,40,41,42,43,44,45,46,47,48,49,50,51,53], of which 15 were considered for a meta-analysis [25,36,37,38,39,40,42,43,44,45,46,47,48,49,50,51] (Figure 1). The PRISMA recommendations were followed to report the results of the review and meta-analysis.

### 3.2. Quality Assessment of Selected Articles

Twenty-one studies satisfied the selection criteria and were included. Sixty-nine-point two percent of the entries were positively reported. All twenty-one studies reported items 1–5, 10, and 11 (abstract, introduction, intervention, outcome, and statistical method). Twenty studies discussed the limitations (item 12) of the study. Nineteen studies stated the source of funding (Item 13), whereas only eleven studies included information regarding the accessibility of the complete trial protocol (Item 14). None of the studies provided any information related to randomization and blinding (Items 6–9).

### 3.3. The Characteristics of the Selected Studies

Twenty out of twenty-one studies were published in the last five years (2021–2025) [25,33,35,36,37,38,39,40,41,42,43,44,45,46,47,48,49,50,51,53], whereas one study was conducted in 2016 [34]. Five studies were conducted in Brazil [25,40,43,49,50], with three each in Saudi Arabia [36,42,53], Spain [41,43,49], and the Republic of Korea [33,45,46]. Additionally, there were two studies each in the United Kingdom [38,39] and Romania [35,47], and one study each in the United States [37], Italy [48], and the Netherlands [34]. Eighteen articles evaluated and discussed only the mechanical properties [25,34,35,36,37,38,39,41,42,43,45,46,47,48,49,50,51,53], one discussed only the physical properties [44], whereas two articles discussed both the physical and mechanical properties [33,40]. In most articles, the tested printing orientations were limited to 0°, 45°, and 90°. Most of the articles tested these properties on specimens of rectangular, cylindrical, or bar-shaped objects. Anatomical crowns were used in five studies [36,42,45,51,53] to test specific properties. For better understanding, separate tables were used to tabulate the results of each tested physical and mechanical property (Table 3, Table 4, Table 5, Table 6, Table 7, Table 8, Table 9, Table 10, Table 11, Table 12 and Table 13).

### 3.4. Assessment of Strength of Evidence

To evaluate the certainty of the evidence from the included studies, the Grading of Recommendations Assessment Development and Evaluation (GRADE) approach was employed [78]. The five domains were: Inconsistency, Indirectness, Imprecision, Risk of Bias, and Publication Bias. The certainty levels vary between very low, low, moderate, and high. In the present review, the selected studies reported a moderate level of certainty of evidence (Table 14).

### 3.5. The Results of Studies Investigating the Mechanical and Physical Properties

#### 3.5.1. Microhardness

**Figure 2:**
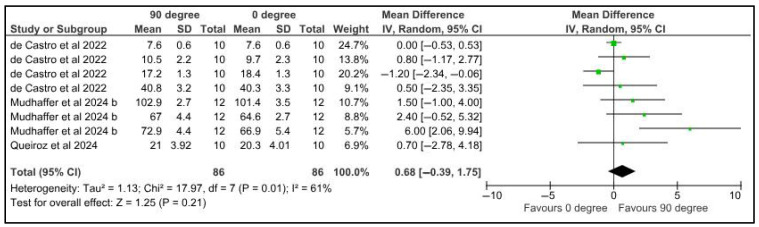
Forest plot: Comparison of microhardness between polymeric specimens printed at 90° and 0° (de Castro et al., 2022 [25], Mudhaffer et al., 2024 b [39]; Queiroz et al., 2024 [43]).

Three studies analyzed and compared the microhardness of various provisional resins printed at different orientations [25,39,43]. A total of eight provisional resin materials were evaluated. A study by Queiroz et al. [43] reported the highest microhardness for specimens printed at 45°, followed by those printed at 90° and 0°. Similar results were reported by de Castro et al. [25] for Nanolab 3D resin and Mudhaffer et al. [39] for Nextdent CB MFH resin. For the Cosmos Temp-DLP [25], Dima CB temp [39], and GC temp print [39] resins, the specimens printed at 90° displayed higher microhardness, followed by specimens printed at 45 ° and 0°. All the tested 3D-printed resins (irrespective of the printing angulation) displayed lower microhardness than milled provisional resins. The data for the meta-analysis comparing microhardness was made available by three Queiroz et al. [43] Microhardness was compared among provisional restorations with different printing orientations (90° and 0 °). Three studies provided eight datasets for meta-analysis. The pooled results of 86 samples showed a mean difference of 0.68 MPa with a confidence interval of 0.39 to 1.75. There was a significant difference in the microhardness of samples at different printing orientations (*p* = 0.01). There was a high heterogeneity, denoted by the I-square, which was 61%. However, the heterogeneity was not statistically significant, with a *p*-value of 0.21 (Figure 2).

**Figure 3:**
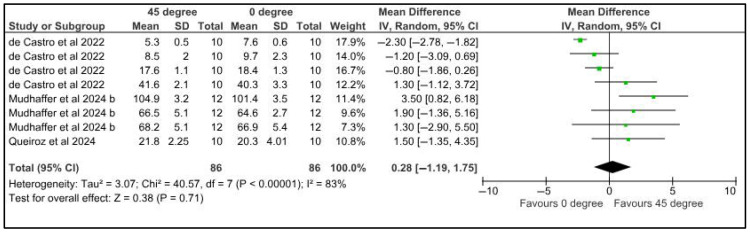
Forest plot: Comparison of microhardness between polymeric specimens printed at 45° and 0° (de Castro et al., 2022 [25], Mudhaffer et al., 2024 b [39]; Queiroz et al., 2024 [43]).

Microhardness was compared among provisional restorations with different printing orientations, 45° and 0°. Three studies provided eight datasets for meta-analysis. The pooled results of 86 samples showed a mean difference of 0.28 MPa with a confidence interval of 1.19 to 1.75. There was a significant difference in the microhardness of samples at different printing orientations (*p* < 0.00001). There was a high heterogeneity, denoted by the I-square, which was 83%. However, the heterogeneity was not statistically significant, with a *p*-value of 0.71 (Figure 3).

#### 3.5.2. Fracture Resistance

Two articles compared the fracture resistance of various provisional resins printed at different orientations [36,42]. A total of three provisional resin materials were evaluated. A study by Alkhateeb et al. [46] reported the highest fracture resistance for specimens printed at 45°, followed by those printed at 0° and 90°. Meanwhile, a study by Aljehani et al. [36] reported the highest fracture resistance for specimens printed at 90°, followed by those printed at 0° and 45°.

**Figure 4:**
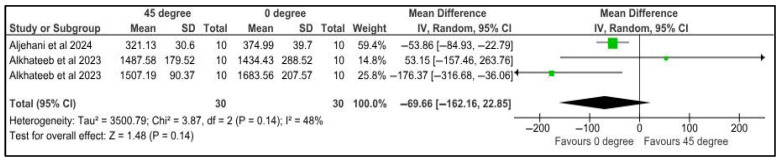
Forest plot: Comparison of fracture strength between polymeric specimens printed at 45° and 0° (Aljehani et al., 2024 [36]; Alkhateeb et al., 2023 [42]).

Fracture strength was compared among provisional restorations with different printing orientations, 0° and 45°. Two studies provided three datasets for meta-analysis. The pooled results of 30 samples showed a mean difference of 69.66 Newtons with a confidence interval of 162.16 to 22.85. There was no significant difference in the fracture strength of samples at different printing orientations (*p* = 0.14). There was a high heterogeneity, denoted by the I-square, which was 48%. However, the heterogeneity was not statistically significant, with a *p*-value of 0.14 (Figure 4).

**Figure 5:**
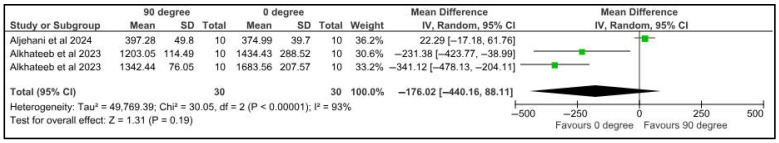
Forest plot: Comparison of fracture strength between polymeric specimens printed at 90° and 0°v (Aljehani et al., 2024 [36]; Alkhateeb et al., 2023 [42]).

Fracture strength was compared among provisional restorations with different printing orientations, 0° and 90°. Two studies provided three datasets for meta-analysis. The pooled results of 30 samples showed a mean difference of 176.02 Newtons with a confidence interval of 440.16 to 88.11. There was a significant difference in the fracture strength of samples at different printing orientations (*p* < 0.00001). There was a high heterogeneity, denoted by the I-square, which was 93%. However, the heterogeneity was not statistically significant, with a *p*-value of 0.19 (Figure 5).

#### 3.5.3. Surface Roughness

Six studies analyzed and compared the surface roughness of various provisional resins printed at different orientations [37,40,43,49,51,53]. A study by Queiroz et al. [43] reported the highest surface roughness for specimens printed at 0°, followed by those printed at 90° and 45°. Four materials reported higher surface roughnesses when printed at 45° followed by 90° and 0° [37,40,49,51]. For the two materials, Cosmos Temp SLA and Cosmos Temp DLP [40], surface roughness was higher for specimens printed at 90°, followed by 45° and 0°.

**Figure 6:**
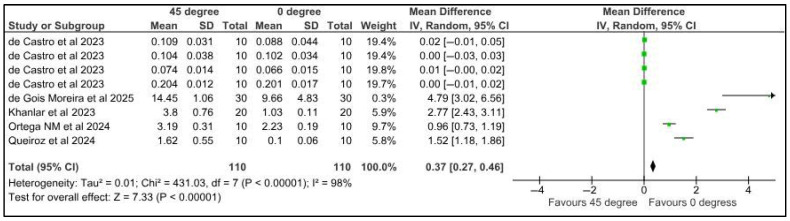
Forest plot: Comparison of surface roughness between polymeric specimens printed at 45° and 0° (de castro et al., 2023 [25]; de Gois Moreira et al., 2025 [49]; Khanlar et al., 2023 [37]; Ortega et al., 2024 [51]; Queiroz et al. [43]).

Five studies provided data for the meta-analysis to compare the microhardness of specimens printed at different orientations. Surface roughness was compared among provisional restorations with different printing orientations, 0° and 45°. Five studies provided eight datasets for meta-analysis. The pooled results of 110 samples showed a mean difference of 0.37 µm with a confidence interval of 0.27 to 0.46. There was a significant difference in the surface roughness of samples at different printing orientations (*p* < 0.00001). There was a high heterogeneity, denoted by the I-square, which was 98%. However, the heterogeneity was statistically significant, with a *p*-value of <0.00001. (Figure 6).

**Figure 7:**
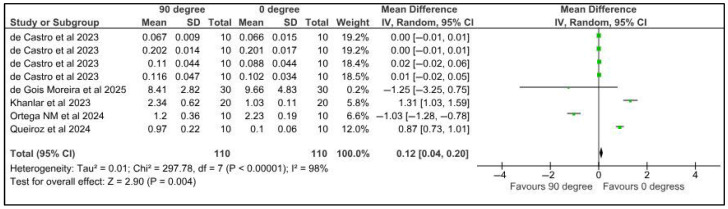
Forest plot: Comparison of surface roughness between polymeric specimens printed at 90° and 0° (de castro et al., 2023 [25]; de Gois Moreira et al., 2025 [49]; Khanlar et al., 2023 [37]; Ortega et al., 2024 [51]; Queiroz et al. [43]).

Five studies provided data for the meta-analysis to compare the microhardness of specimens printed at different orientations. Surface roughness was compared among provisional restorations with different printing orientations, 0° and 90°. Five studies provided eight datasets for meta-analysis. The pooled results of 110 samples showed a mean difference of 0.12 µm with a confidence interval of 0.04 to 0.20. There was a significant difference in the surface roughness of samples at different printing orientations (*p* < 0.00001). There was a high heterogeneity, denoted by the I-square, which was 98%. However, the heterogeneity was statistically significant, with a *p*-value of <0.004 (Figure 7).

#### 3.5.4. Wear Resistance/Wear Volume Loss

Two studies analyzed and compared the wear volume loss of various provisional resins printed at different orientations [45,46]. Lee et al. [45] reported the highest wear volume loss for samples printed at 90°, followed by those printed at 45° and 0°. Whereas Wan et al. [46] reported the highest wear volume loss for samples printed at 0°, followed by those printed at 90° and 45°.

**Figure 8:**
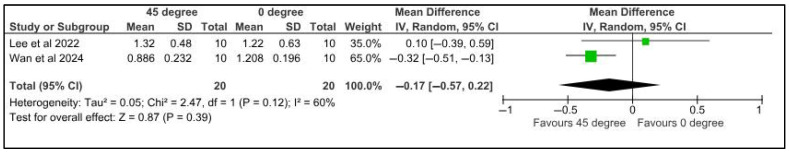
Forest plot: Comparison of wear volume loss between polymeric specimens printed at 45° and 0° (Lee et al., 2022 [33]; Wan et al., 2024 [46]).

Two studies provided data for the meta-analysis to compare the wear volume loss of specimens printed at different orientations. Wear volume loss was compared among provisional restorations with different printing orientations, 0° and 45°. Two studies provided two datasets for meta-analysis. The pooled results of 20 samples showed a mean difference of 0.17 mm^3^ with a confidence interval of 0.57 to 0.22 mm^3^. There was no significant difference in wear volume loss between samples at different printing orientations (*p* = 0.12). There was a high heterogeneity, denoted by the I-square, which was 60%; however, the heterogeneity was statistically non-significant (*p* = 0.39) (Figure 8).

**Figure 9:**
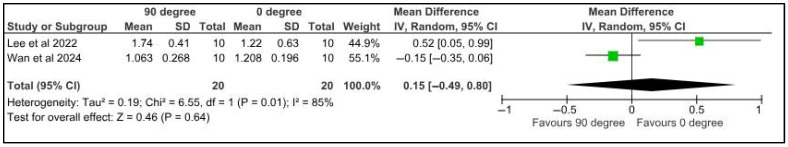
Forest plot: Comparison of wear volume loss between polymeric specimens printed at 90° and 0° (Lee et al., 2022 [33]; Wan et al., 2024 [46]).

Two studies contributed to the data for the meta-analysis comparing the wear volume loss of specimens printed at various orientations. Wear volume loss was compared among provisional restorations with different printing orientations, 0° and 90°. Two studies provided two datasets for meta-analysis. The pooled results of 20 samples showed a mean difference of 0.15 mm^3^ with a confidence interval of 0.49 to 0.80. There was a significant difference in the wear volume loss of samples at different printing orientations (*p* = 0.01). There was a high heterogeneity, denoted by the I-square, which was 85%. However, the heterogeneity was statistically non-significant (*p* = 0.12) (Figure 9).

#### 3.5.5. Flexural Strength

Seven studies analyzed and compared the flexural strength of various provisional resins printed at different orientations [25,38,43,47,48,49,50]. Different materials reported varied results related to the effect of printing orientation on flexural strength.

Five studies contributed to the data for the meta-analysis comparing the flexural strength of specimens printed at different orientations.

**Figure 10:**
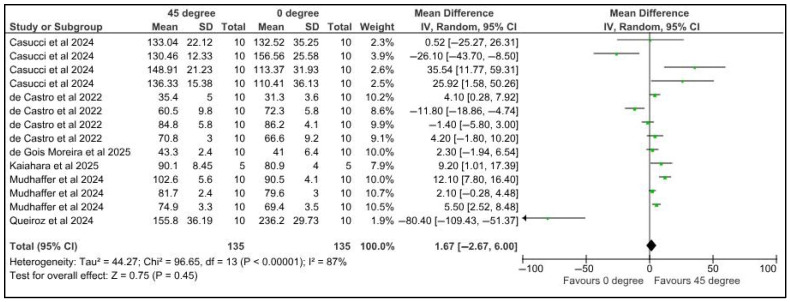
Forest plot: Comparison of flexural strength between polymeric specimens printed at 45° and 0° (Casucci et al., 2024 [48]; de Castro et al., 2022 [25]; de Gois Moreira et al., 2025 [49]; Kaiahara et al., 2025 [50]; Mudhaffer et al., 2024 [38]; Queiroz et al., 2024 [43]).

Flexural strength was compared among provisional restorations with different printing orientations, 0° and 45°. Five studies provided 14 datasets for meta-analysis. The pooled results of 135 samples showed a mean difference of 1.67 Newtons with a confidence interval of 2.67 to 6. There was a significant difference in the flexural strength of samples at different printing orientations (*p* < 0.00001). There was a high heterogeneity, denoted by the I-square, which was 87%. However, the heterogeneity was not statistically significant, with a *p*-value of 0.45 (Figure 10).

**Figure 11:**
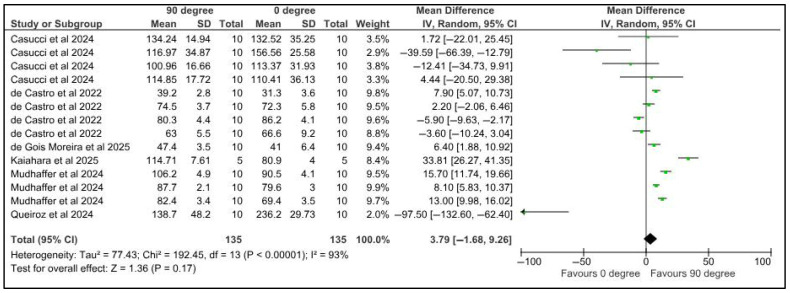
Forest plot: Comparison of flexural strength between polymeric specimens printed at 90° and 0° (Casucci et al., 2024 [48]; de Castro et al., 2022 [25]; de Gois Moreira et al., 2025 [49]; Kaiahara et al., 2025 [50]; Mudhaffer et al., 2024 [38]; Queiroz et al., 2024 [43]).

Flexural strength was compared among provisional restorations with different printing orientations, 0° and 90°. Five studies provided 14 datasets for meta-analysis. The pooled results of 135 samples showed a mean difference of 3.79 Newtons with a confidence interval of 1.68 to 9.26. There was a significant difference in the flexural strength of samples at different printing orientations (*p* < 0.00001). There was a high heterogeneity, denoted by the I-square, which was 93%. However, the heterogeneity was not statistically significant, with a *p*-value of 0.17 (Figure 11).

#### 3.5.6. Color Change

Three studies analyzed and compared the change in color of provisional resins printed at different orientations [33,40,44]. Two studies reported that color change is influenced by print orientation [33,44], whereas one study [40] reported that printing orientation has no influence on color change. Lee et al. [33] reported that the highest color change was observed in specimens printed at 90°, followed by those printed at 45° and 0°.

#### 3.5.7. Tensile Strength, Compressive Strength, and Elastic Modulus Results

One study analyzed and compared the tensile strength of provisional resins printed at different orientations [35]. The study reported that tensile strength is influenced by print orientation. When printed at a 50 μm layer thickness, the highest tensile strength was reported for specimens printed at 0°, followed by those printed at 90° and 45°. Whereas, when printed at a 100 μm layer thickness, the highest tensile strength was reported for specimens printed at 0°, followed by those printed at 45° and 90°.

One study analyzed and compared the elastic modulus of four provisional resins printed at different orientations [41]. The study reported that the elastic modulus is not significantly influenced by print orientation. For Detax Freeprint temp and Formlabs Temporary CB, the elastic modulus was higher when printed at 90°, followed by 0°. Whereas, for GCT-GC, the elastic modulus was higher when printed at 0°, followed by 90°.

Two studies analyzed and compared the compressive strength of provisional resins printed at different orientations [34,35]. The studies reported that compressive strength is influenced by print orientation. For both studies, the compressive strength was reported to be higher for specimens printed at 90° compared to those printed at 0°.

## 4. Discussion

The present systematic review and meta-analysis included all available studies that evaluated and compared the physical and mechanical properties of 3D-printed provisional restorative materials printed at different orientations. The PRISMA recommendations were followed to configure and organize this section of the review and meta-analysis. A total of twenty-one research articles were included in this study [25,33,34,35,36,37,38,39,40,41,42,43,44,45,46,47,48,49,50,51,53]. The overall findings of the present review suggest that printing orientations influence the physical and mechanical properties of 3D-printed provisional restorative materials. The extent of the variation varies according to the type of material and the tested property. Therefore, the tested null hypothesis was rejected.

Any provisional fixed dental prosthesis present in the oral cavity must withstand a variety of forces that may lead to its failure [79,80]. Thus, it is vital to fabricate these prostheses in a manner that meets the minimum required standards to resist distortion and subsequent failure under multiple oral forces. A review and meta-analysis by Jain et al. [20] reported that 3D-printed provisional crowns and fixed dental prosthesis are acceptable alternatives to milled and conventional provisional materials and exhibit superior mechanical but inferior physical properties when compared to milled and conventional provisional materials. The mechanical and physical properties discussed in the present review and meta-analysis are fracture strength, tensile strength, compressive strength, microhardness, surface roughness, wear resistance, flexural strength, elastic modulus, and color stability.

Alkhateeb et al. [42] reported higher fracture strengths for specimens printed at 0° and 45°. In contrast, Aljehani et al. [36] reported contrasting results. Similar findings were reported by Turksayar et al. [81]. The higher fracture strength observed in the specimens printed at 0° could be related to the load direction with respect to the printing layer. When the load was applied to specimens printed at 90°, the splitting of a few specimens along the printing layer direction was observed due to poor interlayer bonding compared to bonding within the layer [42,81,82].

When microhardness was evaluated, Queiroz et al. [48], Mudhaffer et al. [39], and de Castro et al. [25] reported no significant effect of printing orientation on microhardness. This lack of an effect was attributed to the homogeneity of 3D-printed specimens [25] or the same polymerization process of the groups, as the light application that occurs during printing layers is not influenced by printing orientation [43]. Alaqeel et al. [83] reported a lower hardness of occlusal splints printed at an orientation of 0° compared to a 90° orientation. They attributed this to the presence of micropores in the 0° print orientation specimens as observed in SEM images.

Studies reporting the effect of printing orientation on flexural strength have varied outcomes. Durban et al. [47] and Queiroz et al. [43] reported a higher flexural strength for specimens printed at 0°. Contrary to this, Mudhaffer et al. [38], Espinar et al. [41], and Kaiahara et al. [50] reported higher flexural strengths for specimens printed at 90°. Studies by Casucci et al. [48] and de Castro et al. [25], which involved different resin materials, reported varied results depending on the material used. Lower flexural strengths for 90° printed specimens were attributed to the direction of the load being parallel to the printing layer, which may lead to the splitting of the layers due to weaker strength between successive layers compared to that within individual layers [9,34,38,84]. Whereas, higher flexural strengths for 90° printed specimens were attributed to strong adhesion between layers, making strength differences negligible [15,23]; different degrees of conversion during polymerization [15,38]; different light exposures influencing the degree of conversion [15,38]; differences in the filler content of different tested materials, as higher filler contents improve mechanical properties [85]; and to differences in curing devices and curing times for each tested material [38,86,87].

Lee et al. [45] and Wan et al. [46] stated that specimens printed at a 45° angle have the highest wear resistance. However, Lee et al. [45] reported higher wear resistances for 0° printed specimens, whereas Wan et al. [46] reported higher wear resistances for 90° printed specimens. The higher wear resistances of specimens printed at 0° and 45° were correlated with weak interlayer bonds [45,88]. They suggested that a reduction in printing angle causes a reduction in the wear. Wan et al. [46] related the higher wear resistance of 45° printed specimens to their stepwise surface pattern and their microstructure. They also reported that there is no significant difference in the wear resistance between resins printed with layer thicknesses of 50 and 100 µm.

Khanlar et al. [37], de Gois Moreira et al. [49], Ortega et al. [51], and de Castro et al. [40] described the higher surface roughness of resins printed at 45° compared to those printed at 90° or 0°. De Castro et al. [40] tested four different 3D printing resins and reported various results. Most studies have reported that surface roughness is affected by changes in printing orientation. The high surface roughness of specimens printed at a 45° printing orientation may be due to stepwise linking between layers, where these step edges between layers induce a high surface roughness. [9,37]. Khanlar et al. [37] reported the lowest surface roughness for specimens printed at 0°. Revilla-Leon et al. [89] reported the least surface roughness for silicone aligners printed at 0°, followed by 90° and 45°. Ortega NM et al. [51] reported the lowest surface roughness for specimens printed at a 90° angle. Other factors that can affect surface roughness values include the type of resin, the filler content, the type of printer, the printing technology, the printing layer thickness, and the post-processing process [12,51,90].

Three studies evaluated the effect of printing orientation on the color stability of provisional resins. One study reported that printing orientation does not influence color change [40], whereas two studies [33,44] reported that printing orientation affects color stability. Lee et al. [33] reported the maximum color change in specimens printed at 90°, followed by 45° and 0°. They also reported that color stability is affected by layer thickness, with specimens printed at 100 µm exhibiting less color change than those printed at 25 µm. These findings could be attributed to differences in wettability, the contact angle of a liquid, the dissolution of components of the resins, or differences in the degree of conversion across the thickness of the specimens [40,44,91].

### Strengths and Limitations

In this review, all articles discussing the effect of printing orientation on 3D-printed provisional resin materials were reviewed and subsequently selected based on predefined criteria. The detailed and comprehensive search strategy, along with unbiased assessments of the articles by the reviewers, are the main strengths of this review. The author proposes that further in vivo studies should be performed with larger specimen numbers to facilitate better evaluation under clinical scenarios, along with the use of standardized techniques for specimen fabrication and testing to reduce variability, and the presentation of results in both graphical and tabular forms for easy data extraction by readers [92]. Limitations include the high risk of bias and high data variability in the studies included. The use of different types of resins, printing machines, technologies, and specimen fabrication techniques increased the variability. Another limitation is the heterogeneity of the testing parameters across the included in vitro studies. Authors should follow standardized protocols established by the American Dental Association (ADA) or the International Organization for Standardization (ISO) for the fabrication and testing of specimens, as this will enhance the generalizability of the results. Additionally, whenever feasible, authors should report the results in both tabular and graphical form for the ease of use for further research. High heterogeneity was observed in most meta-analyses, and the majority of the pooled estimates yielded inconclusive results. In the present review, the included studies are predominantly in vitro in nature, which may not simulate the actual oral conditions and thus may not accurately predict clinical performance, and may limit the applicability of the results to clinical settings. More in vivo studies should be conducted to guide dentists and dental technicians in developing protocols and setting parameters for the printing of provisional resin materials to achieve the best clinical outcomes. The present review focused only on physical and mechanical properties. Other parameters, such as internal adaptation, marginal fit, accuracy, and trueness, which are important in material selection, should also be studied in further systematic reviews.

## 5. Conclusions

Within the limitations of this review and meta-analysis, the following conclusions can be drawn: Printing orientation affects some of the tested properties, which include fracture strength (significantly higher for specimens printed at 0° when compared to 90°), wear resistance (significantly higher for specimens printed at 90° when compared to 0°), microhardness (significantly higher for specimens printed at 90° and 45° when compared to 0°), color stability (high at 0°), and surface roughness (significantly higher for specimens printed at 45° and 90° when compared to 0°). There were varied outcomes in terms of flexural strength and elastic modulus. Further in vivo studies should be conducted to explore the relationship between printing orientation and the properties of provisional resins, which can help in the development of protocols and the setting of parameters for the best clinical outcomes. Researchers should adhere to strict blinding protocols in their studies to minimize bias and enhance quality.

## Figures and Tables

**Table 1:** Selection criteria.

Inclusion Criteria	Exclusion Criteria
Research articles in the English language	Research articles in a language other than English.
Human clinical studies; in vitro studies	Animal studies.
Studies comparing the influence of printing orientation on the physical and mechanical properties of 3D-printed polymeric provisional dental restorations	Editorials, case reports, theses, incomplete trials, reports, commentaries, review papers, conference papers, unpublished abstracts.
	Studies comparing the influence of printing orientation on the marginal or internal fit of 3D-printed provisional dental restorations.
	Studies comparing the influence of printing orientation on the other properties of 3D-printed provisional dental restorations.
	Studies comparing the effect of printing orientation on materials other than provisional dental resins (for example, ceramics or metallic alloys).
	Studies comparing the effect of printing orientation on various properties of 3D-printed resins used for the fabrication of denture bases, dies, models, or orthodontic aligners.
	Studies comparing properties of materials under trial.

**Table 2:** Characteristics and summary of the articles.

Author and Year	Studied Characteristics	Reviewed Property	Sample Size (*n*)	Details of the Assessed Materials	Primary Composition	Printing Orientation	Form and Size of Tested Specimens	Layer Thickness	Printer Type/Brand/ Tradename
Alharbi et al., 2016 [34], Netherlands	Compressive strength	MP	*n* = 40 (20/orientation)	Temporis (DWS)	Hybrid composite resin	(a) 0° (b) 90°	Cylinder (height: 5.04–4.97 mm, diameter: 3.07 mm)	50 μm	DW028D three-dimensional printer
Derban et al., 2021 [47], Romania	Flexure Strength	MP	*n* = 60 (30 material) (10 orientation)	(A) DETAX Freeprint Temp (Detax Gmbh & Co., Ettlingen, Germany); (B) NEXTDENT C & B MFH (Vertex Dental B.V., Soesterberg, The Netherlands)	(A) Methylmethacrylates; (B) Micro filled hybrid methacrylic oligomers	(a) 0° (b) 45° (c) 90°	Parallelepiped shaped (25 mm × 2 mm × 2 mm)	50 μm	SheraPrint D30 printer (Shera Material Technology Gmbh, Lemforde, Germany)
Alharbi et al., 2021 [53], Saudi Arabia	Surface roughness	MP	*n* = 45 (9/orientation)	Temporis A2 (DWS systems)	Hybrid composite resin	(a) 90 (b) 120 (c) 135 (d) 150 (e) 180	Anatomical crown maxillary central incisor	50 µm	028DWS SLA printer (DWS systems)
Lee at al., 2022 [45], Korea	Wear volume	MP	*n* = 20 (5 patients) (1 orientation and 1 conventional resin)	(A) Conventional self-cure resin: Unifast III (GC Corporation, Seoul, Republic of Korea)); (B) 3D-printed resin: RAYDENT C&B (Ray Co., Ltd., Hwaseong-si, Republic of Korea)	(A) Methylmethacrylate resin; (B) hybrid composite resin	(a) 0° (b) 45° (c) 90°	Crown (Molar)	50 μm	MEG-PRINTER 3D II (Megagen, Daegu, Republic of Korea)
de Castro et al., 2022 [25], Brazil	Flexural strength; flexural modulus; microhardness	MP	For FS and FM *n* = 1430 (30 materials, 10 orientation, and 10 milled)	(A) Cosmos Temp SLA (Yller, Pelotas, RS, Brazil) (B) Cosmos Temp DLP (Yller, Pelotas, RS, Brazil) (C) PriZma Bioprov (Makertech, Tatuí, SP, Brazil) (D) NAnolab 3D (Wilcos do Brasil, Petr´ opolis, RJ, Brazil)	(A) and (B): Oligomers, monomers (C) acrylic monomers and oligomers; (D) nanohybrid resin	(a) 0° (b) 45° (c) 90°	FS: Bar (25 × 2 × 2 mm) microhardness analyses: disk (15 mm in diameter and 2.5 mm thick)	50 μm	(A) FormLabs 2 (FormLabs, Somerville, MA, USA) (B) P30 (Straumann Basel, Switzerland) (C) Flash Forge Hunter (Zhejiang Flashforge 3D technology Co., Jinhua City, ZJ, China) (D) W3D (Wilcos do Brasil, Petr´opolis, RJ, Brazil)
Lee et al., 2022 [33], South Korea	Color stability; roughness	PP MP	*n* = 180 (90 layer thickness) (30 orientation)	C&B 5.0 Hybrid; ARUM	Urethane Dimethacrylate	(a) 0° (b) 45° (c) 90°	Disk-shaped (15 × 3 mm)	25 μm 100 μm	ASIGA MAX UV (ASIGA NSW, Australia)
de Castro et al., 2023 [40], Brazil	Roughness; color	MP PP	For FS and FM *n* = 130 (30 materials, 10 orientation, and 10 milled)	(A) Cosmos Temp SLA (Yller, Pelotas, RS, Brazil); (B) Cosmos Temp DLP (Yller, Pelotas, RS, Brazil); (C) PriZma Bioprov (Makertech, Tatuí, SP, Brazil); (D) NAnolab 3D (Wilcos do Brasil, Petr´ opolis, RJ, Brazil); (E) Vita CAD-Temp (Vita Zahnfabrik, Bad Säckingen, Germany) (Control)	(A) and (B): Formlabs Temporary CB; (C) acrylic monomers and oligomers; (D) nanohybrid resin; (E) PMMA	(a) 0° (b) 45° (c) 90°	Disks (15 mm in diameter, 2.5 mm in thickness)	50 μm	(A) FormLabs 2 (FormLabs, Somerville, MA, USA) (B) P30 (Straumann Basel, Switzerland) (C) Flash Forge Hunter (Zhejiang Flashforge 3D technology Co., Jinhua City, ZJ, China)) (D) W3D (Wilcos do Brasil, Petr´opolis, RJ, Brazil)
Farkas et al., 2023 [35], Romania	Tensile strength; compression strength	MP	For tensile strength: *n* = 24 Layer thickness 100 μm: *n* = 12 50 μm: *n* = 12 (4 orientation) For compression test *n* = 12 (layer thickness 100 μm) (4 orientation)	NextDent C&B MFH (3D Systems, Rock Hill, SC, USA) (ND)	Micro filled hybrid methacrylic oligomers	(a) 0° (b) 45° (c) 90°	For tensile strength: Bar (NM) For compression test: Cylinder (NM)	For tensile strength: 100 μm and 50 μm; For compression test: 50 μm	ANYCUBIC Photon Mono X
Alkhateeb et al. [42], 2023, Saudi Arabia	Fracture load	MP	*n* = 300 (150 resin, *n* = 10 orientation, 10 post-curing time)	(A) NextDent, C&B (NextDent, Soesterberg, The Netherlands) (CB); (B) ASIGA Asiga DentaTOOTH (ASIGA, Erfurt, Germany)	(A) Micro filled hybrid methacrylic oligomers; (B) Methacrylate-based microhybrid composite resin	(a) 0° (b) 45° (c) 90°	Three-unit interim FDP	50 μm	NextDent 5100 DLP (3D Systems, Rock Hill, SC, USA) ASIGA MAX LED-based DLP (Asiga, Alexandria, Australia)
Espinar et al., 2023 [44], Spain	Color stability	PP	*n* = 24 (3 orientation)	(A) Detax Freeprint Temp (DETAX GmbH, Ettlingen, Germany); (B) Formlabs Temporary CB (Formlabs Inc., Somerville, MA, USA); (C) Formlabs Permanent Crown (Formlabs Inc., Somerville, MA, USA); (D) GCT- GC TempPrint, (GC Corporation, Tokyo, Japan)	(A) Methylmethacrylates; (B) Formlabs Temporary CB; (C) Ceramic-filled resin; (D) UDMA	(a) 0° (b) 90°	Square-shaped specimens (10 mm × 10 mm × 1.2 mm)	50 µm	Asiga Max UV1 (Asiga, Alexandria, Australia)
Espinar et al., 2024 [41], Spain	Flexural strength; elastic modulus	MP MP	*n* = 160 (40 material, 20 orientation)	(A) Detax Freeprint Temp (DETAX GmbH, Ettlingen, Germany); (B) Formlabs Temporary CB (Formlabs Inc., Somerville, MA, USA); (C) Formlabs Permanent Crown (Formlabs Inc., Somerville, MA, USA); (D) GCT- GC (TempPrint, (GC Corporation, Tokyo, Japan)	(A) Methylmethacrylates; (B) Formlabs Temporary CB; (C) Ceramic-filled resin; (D) UDMA	(a) 0° (b) 90°	Bar-shaped specimens (25 mm × 2 mm × 2 mm)	50 µm	Asiga Max UV1 (Asiga, Alexandria, Australia)
Queiroz et al., 2024 [43], Brazil	Flexural strength; microhardness; surface roughness	MP	*n* = 30 (10 orientation)	AA Temp, PrintaX, (Odonto Mega import, Odonto Mega import, Ribeirão Preto, SP, Brazil)	N/M	(a) 0° (b) 45° (c) 90°	Bar (4 × 2 × 10 mm)	50 μm	Mikra Resin 3D Printer (Zhangzhou Echo Technology Co., Ltd., Zhangzhou, China)
Mudhaffer et al., 2025 [38], United Kingdom	Flexural strength; flexural modulus	MP	*n* = 540 (180 material) (60 orientation)	(A) Nextdent CB MFH (3D systems, Netherlands) (ND) (B) Dima CB temp (Kulzer GmbH, Germany) (DT) (C) GC temp print (GC dental, Japan) (GC)	(A) Microfilled Hybrid Methacrylic oligomers; (B) Esterification products of isopropylidiphenol; (C) UDMA	(a) 0° (b) 45° (c) 90°	Bar (2 × 2 × 25 mm)	50 μm	ASIGA MAX UV; (ASIGA, NSW, Australia)
Casucci et al., 2024 [48], Italy	Flexural strength	MP	*n* = 120 (30 material) (10 orientation)	(A) Varseo smile teeth (VS) (Bego GmbH & Co., Bremen, Germany) (B) V-print C&B temp (VP) (Voco GmbH, Cuxhaven, Germany) (C) Bego Triniq (BT) (Bego GmbH & Co., Bremen, Germany) (D) Sprintray Crown (SC) (SprintRay, CA, USA)	(A) Isopropylidenediphenol; (B)UDMA Bis-EMA TEGDMA; (C) methylprop-2enoic acid, alpha-oxo-methyl ester; (D) methylbenzoateformate	(a) 0° (b) 45° (c) 90°	Bar (25 × 2 × 2 mm)	50 μm	ASIGA MAX UV; (ASIGA, NSW, Australia)
Wan et al., 2024 [46], South Korea	Wear resistance	MP	*n* = 60 (10 orientation; 10 layer thickness)	C&B MFH (NextDent (3D systems, The Netherlands)	Methacrylic oligomers	(a) 0° (b) 45° (c) 90	Rectangular specimens (15 × 10 × 10 mm)	50 µm 100 µm	MAX UV, (Asiga, NSW, Australia)
Ortega NM et al., 2024 [51], Spain	Surface roughness	MP	*n* = 30 (10/orientation)	GC Temp PRINT (GC Corporation, Tokyo, Japan)	UDMA	(a) 0° (b) 45° (c) 90	Anatomical crown maxillary right premolar	50 µm	Asiga MAX UV (Asiga, NSW, Australia)
Aljehani et al., 2024 [36], Saudi Arabia	Fracture resistance	MP	*n* = 40 (10 orientation and 10 milled)	(A) Freeprint temp (Detax, Ettlingen, Germany); (B) Milled (control group): Coratemp, (White Peaks, Germany)	(A) Methylmethacrylates; (B) polymethyl methacrylate	(a) 0° (b) 45° (c) 90°	Fully contoured anatomical crown (central incisor)	50 μm	Asiga Max, (Asiga, NSW, Australia)
Khanlar et al., 2023 [37], United States	Surface roughness	MP	*n* = 80 (20 orientation and 20 conventional)	(A) E-Dent C&B MHF (EnvisionTEC Inc, GmbH, Gladbeck, Germany); (B) Protemp 4 (3M ESPE)	(A) Microfilled hybrid material; (B) Bis-acryl resin	(a) 0° (b) 45° (c) 90°	Disk-shaped specimens (20 × 10 mm)	NM	Envisiontec VIDA HD; (EnvisionTEC GmbH, Gladbeck, Germany)
Mudhaffer et al., 2024 [39], United Kingdom	Martens hardness	MP	3D-rinted *n* = 108 (36 material) (12 orientation); Milled (*n* = 24) (12 material)	3D-Printed: (A) Nextdent CB MFH, 3D systems, The Netherlands (ND); (B) Dima CB temp, Kulzer, Germany (DT); (C) GC temp print, GC dental, Japan (GC) Milled; (D) LAVA ultimate, 3 M ESPE, USA; (E) Telio CAD, Ivoclar vivadent AG	(A) Microfilled Hybrid Methacrylic oligomers; (B) Esterification products of isopropylidiphenol; (C) UDMA; (D) BisGMA, UDMA; (E) PMMA	(a) 0° (b) 45° (c) 90°	Disks 20 mm (diameter) × 2.3 mm (height)	50 μm	ASIGA MAX UV; (ASIGA, NSW, Australia)
de Gois Moreira et al., 2025 [49], Brazil	Flexural strength Roughness	MP	For Flexural strength: *n* = 450 (150 layer thickness) (30 orientation); For roughness *n* = 2 groups	Cosmos Tempo (Yller Biometeriais SA, Pelotas, Brazil)	Oligomers; monomers	(a) 0° (b) 30° (c) 45° (d) 60° (e) 90°	Bars (26 mm × 2.2 mm × 2.2 mm)	25 μm 50 μm 100 μm	Forms 2, Formslab
Kaiahara et al., 2025 [50], Brazil	Flexural strength	MP	*n* = 15 (5/orientation) (*n* = 5; control group)	COSMOS TEMP, A1, (Yller Biomaterials, Pelotas, Brazil); Milled resin: Duralay color 81(Reliance Dental MFG Co, IL, USA)	Methacrylates PMMA	(a) 0° (b) 45° (c) 90	Bar-shaped specimen	50 µm	BASIC PRINTER X (3DBasic, Marília, Sao Paulo, Brazil)

MP: Mechanical Property; PP: Physical Property.

**Table 3:** Fracture strength outcomes.

Author and Year	Alkhateeb et al., 2023 [42]	Aljehani et al., 2024 [36]
Maximum Fracture Force (N) (Printing angle: 0°)	Fracture Load: *(A) NextDent, C&B:* Post-curing time: 0 min: 610.06 ± 208.95; 30 min: 980.72 ± 298.70; 60 min: 1259.64 ± 205.80; 90 min: 1476.99 ± 71.47; 120 min: 1683.56 ± 207.57 *(B) ASIGA resin:* Post-curing time: 0 min: 794.83 ± 68.52; 30 min: 1013.31 ± 140.13; 60 min: 1067.35 ± 75.42; 90 min: 1267.00 ± 240.58; 120 min: 1434.43 ± 288.52	Fracture resistance: 374.99 ± 39.7
Maximum Fracture Force (N) (Printing angle: 45°)	Fracture Load: *(A) NextDent, C&B:* Post-curing time: 0 min: 532.83 ± 109.21; 30 min: 1307.32 ± 88.89; 60 min: 1438.88 ± 209.60; 90 min: 1437.02 ± 230.00; 120 min: 1507.19 ± 90.37 *(B) ASIGA resin:* Post-curing time: 0 min: 626.32 ± 96.41; 30 min: 1113.47 ± 61.84; 60 min: 1102.81 ± 148.05; 90 min: 1327.30 ± 161.96; 120 min: 1487.58 ± 179.52	Fracture resistance: 321.13 ± 30.6
Maximum Fracture Force (N) (Printing angle: 90°)	Fracture Load: *(A) NextDent, C&B:* Post-curing time: 0 min: 503.29 ± 196.37; 30 min: 1168.46 ± 172.91; 60 min: 1207.51 ± 151.98; 90 min: 1237.17 ± 98.03; 120 min: 1342.44 ± 76.05 *(B) ASIGA resin:* Post-curing time: 0 min: 602.03 ± 82.76; 30 min: 1041.01 ± 145.87; 60 min: 1076.02 ± 89.74; 90 min: 1124.87 ± 121.59; 120 min: 1203.05 ± 114.49	Fracture resistance: 397.28 ± 49.8
Maximum Fracture Force (N) for Milled/conventional specimens (N)	N/A	Fracture resistance: Milled: 1157.16 ± 75.0
Exposure Agent/Aging Procedure	Thermal cycling for 5000 cycles	No
Testing Machine	Universal testing machine	Universal testing machine
Conclusions &/or Recommendations	For all respective curing times, Fracture Load: 45° > 0° > 90° Post-curing time has a positive effect on fracture load. Fracture load according to curing time: 120 min > 90 min > 60 min > 30 min > 0 min	Fracture resistance: Milled > 3D printed 3D printed: 90° > 0° > 45°

N/A: not applicable; N: Newtons.

**Table 4:** Color Change (ΔE/ΔE_00_) Outcomes.

Author and Year	de Castro et al., 2023 [40]	Lee et al., 2022 [33]	Espinar et al., 2023 [44]
Immersion media/surface treatment	Toothbrushing wear	Distilled water, coffee solution, and red wine	-
Immersion/exposure duration/aging	10,000 cycles	30 days	-
Mean alteration in color of conventional/milled specimens	2.063	-	-
Mean alteration in color of specimens printed at 0°	(A) Cosmos Temp SLA: 3.14 ^##^; (B) Cosmos Temp DLP: 10.01 ^##^ (C) PriZma Bioprov:3.61 ^##^; (D) NAnolab 3D:8.41 ^##^	ΔE_00_ 25 μm: DW: 3.954 ± 0.107; CS: 4.434 ± 0.057; RW: 8.050 ± 0.557 100 μm: DW: 4.259 ± 0.126: CS: 2.243 ± 0.158: RW: 7.078 ± 0.324	ΔE_00_ DFT showed the greatest color differences between 0° and 90° printing orientation
Mean alteration in color of specimens printed at 45°	(A) Cosmos Temp SLA: 3.58 ^##^; (B) Cosmos Temp DLP:9.46 ^##^ (C) PriZma Bioprov:3.74 ^##^; (D) NAnolab 3D:8.63 ^##^	ΔE_00_ 25 μm: DW: 4.058 ± 0.136; CS: 5.384 ± 0.412 RW: 8.732 ± 0.369 100 μm: DW: 3.781 ± 0.132; CS: 4.669 ± 0.093 RW: 9.038 ± 0.152	-
Mean alteration in color of specimens printed at 90°	(A) Cosmos Temp SLA: 3.07 ^##^; (B) Cosmos Temp DLP:9.68 ^##^ (C) PriZma Bioprov:3.55 ^##^; (D) NAnolab 3D:7.58 ^##^	ΔE_00_ 25 μm: DW: 3.297 ± 0.041; CS: 5.697 ± 0.156 RW: 9.431 ± 0.238 100 μm: DW: 3.457 ± 0.060; CS: 5.428 ± 0.189 RW: 9.197 ± 0.247	ΔE_00_ DFT showed the greatest color differences between 0° and 90° printing orientation
Device used	Spectrophotometer (VITA Easyshade^®^ V, Vita Zahnfabrik)	Spectrophotometer (CM 700d; Konica Minolta)	Spectroradiometer (PR 670—Photo Research)
Authors’ suggestions/conclusions	No influence of printing orientation on the change in color	Printing orientation affects the color stability ΔE_00:_ 90° > 45° > 0°	Building orientation influences the visual color and translucency. ΔE_00_ varies with the type of 3D-printed resin.

##: Data retrieved from plot digitizer app. DW: Distilled water; CS: Coffee solution; RW: Red Wine.

**Table 5:** Tensile Strength (TS) Outcomes.

Author and Year	Farkas et al., 2023 [35]
Mean tensile strength for specimens printed at 0° (MPa)	Layer thickness: 100 μm: 56.81; 50 μm: 58.53
Mean tensile strength for specimens printed at 45° (MPa)	Layer thickness: 100 μm: 51.52: 50 μm: 53.69
Mean tensile strength for specimens printed at 90° (MPa)	Layer thickness: 100 μm: 49.59; 50 μm: 58.01
Exposure agent/aging procedure	No
Testing machine	Universal testing machine
Conclusions and/or suggestions	TS is influenced by print orientation and print layer thickness. TS at 50 μm: 0° > 90° > 45°; TS at 100 μm: 0° > 45° > 90°; TS: 50 μm > 100 μm

**Table 6:** Compressive Strength (CS) Outcomes.

Author and Year	Farkas et al., 2023 [35]	Alharbi et al., 2016 [34]
Mean compressive strength for specimens printed at 0° (Mpa)	Yield stress: 85.90; Max. stress: 146.64	257.7 ± 41.1
Mean compressive strength for specimens printed at 45° (Mpa)	Yield stress: 98.45; Max. stress: 228.28	-
Mean compressive strength for specimens printed at 90° (Mpa)	Yield stress: 110.06; Max. stress: 238.26	297.7 ± 34.4
Exposure agent/aging procedure	No	-
Testing machine	Universal testing machine	Universal testing machine
Conclusions and/or recommendations	CS influenced by print orientation: CS: 90° > 45° > 0°	CS influenced by print orientation: CS: 90° > 0°

**Table 7:** Microhardness Test Outcomes.

Author and Year	Queiroz et al., 2024 [43]	de Castro et al., 2022 [25]	Mudhaffer et al., 2024 [39]
Mean microhardness for specimens printed at 0° (Kgf/mm^2^/KHN/HV/MPa)	VH (HV): 20.30 ± 4.01	Knoop Hardness: Cosmos Temp—SLA: 7.6 ± 0.6 Cosmos Temp—DLP: 9.7 ± 2.3 PriZma BioProv: 18.4 ± 1.3 Nanolab 3D: 40.3 ± 3.3	Martens hardness (A) ND: DW:101.4 ± 3.5; AS: 101.9 ± 4.3 (B) DT: DW: 64.6 ± 2.7; AS: 60.8 ± 5.8 (C) GC: DW: 66.9 ± 5.4; AS: 66.6 ± 5.4
Mean microhardness for specimens printed at 45° (Kgf/mm^2^/KHN/HV/MPa)	VH (HV): 21.80 ± 2.25	Knoop Hardness: Cosmos Temp—SLA: 5.3 ± 0.5 Cosmos Temp—DLP: 8.5 ± 2.0 PriZma BioProv: 18.4 17.6 ± 1.1 Nanolab 3D: 41.6 ± 2.1	Martens hardness (A) ND: DW: 104.9 ± 3.2; AS: 102.4 ± 3.7 (B) DT: DW: 66.5 ± 5.1; AS: 64.4 ± 3.3 (C) GC: DW: 68.2 ± 3.5; AS: 65.3 ± 6.5
Mean microhardness for specimens printed at 90° (Kgf/mm^2^/KHN/HV/MPa)	VH (HV): 21.00 ± 3.92	Knoop Hardness: Cosmos Temp—SLA: 7.6 ± 0.6 Cosmos Temp—DLP: 10.5 ± 2.2 PriZma BioProv: 17.2 ± 1.3 Nanolab 3D: 40.8 ± 3.2	Martens hardness (A) ND:DW: 102.9 ± 2.7; AS: 101.2 ± 4.8 (B) DT: DW: 67.0 ± 4.4; AS: 66.7 ± 3.0 (C) GC: DW: 72.9 ± 4.4; AS: 72.5 ± 2.9
Mean microhardness for specimens fabricated by milling/conventional technique (in MPa)	-	Knoop Hardness: Vita Temp CAD (Control): 28.4 ± 1.8	(D) LAVA ultimate: DW:584.4 ± 14.8; AS: 579.7 ± 9.4 (E) Telio CAD: DW: 119 ± 13.3; AS: 104 ± 12.0
Surface treatment/exposure agent/aging procedure	No	Immersion for 1 year in distilled water	Immersion in distilled water and artificial saliva for 90 days
Surface treatment/exposure agent/aging procedure	HMV-G series (Shimadzu Corp.)	Future-Tech FM Corp.	Zwick Martens Hardness Instrument (Z2.5, ZwickRoell Ltd.)
Authors’ recommendations/conclusions	MH: 45° > 90° > 0°	KH: Cosmos Temp-SLA: 0° = 90° > 45° Cosmos Temp-DLP: 90° > 0° > 45° PriZma BioProv: 0° > 45° > 90° Nanolab 3D: 45° > 90° > 0° Milled > 3D Printed	MH: ND: 45° > 90° > 0° DT: 90° > 45° > 0° GC: 90° > 45° > 0° Milled > 3D Printed

VH: Vickers hardness; HV: Vickers pyramid number; MH: microhardness; KH: Knoop hardness; ND: Nextdent CB MFH; DT: Dima CB temp; GC: GC temp print; DW: Distilled Water.

**Table 8:** Surface Roughness (SR) Test Outcomes.

Author and Year	SR Without Aging/Wear						SR After Aging/Wear			
	Specimens Printed at 0° (Ra/Sa in μm)	Specimens Printed at 30° (Ra/Sa in μm)	Specimens Printed at 45° (Ra/Sa in μm)	Specimens Printed at 60° (Ra/Sa in μm)	Specimens Printed at 90° (Ra/Sa in μm)	Milled/ Conventional Specimens (Ra/Sa in μm)	Specimens Printed at 0° (Ra in μm)	Specimens Printed at 45° (Ra in μm)	Specimens Printed at 90° (Ra in μm)	Milled/ Conventional Specimens (Ra/Sa in μm)
Queiroz et al., 2024 [43]	Ra: 0.10 ± 0.06	-	Ra: 1.62 ± 0.55	-	Ra: 0.97 ± 0.22	NA	NA	NA	NA	
Khanlar et al., 2023 [37]	Sa: 1.03 ± 0.11	-	Sa: 3.80 ± 0.76	-	Sa: 2.34 ± 0.62	Sa: 2.93 ± 0.90	NA	NA	NA	
de Gois Moreira et al., 2025 [49]	Sa: 25 μm: 9.58 ± 3.50 50 μm: 9.66 ± 4.83 100 μm: 16.85 ± 0.64	Sa: 25 μm: 28.25 ± 2.62 50 μm: 10.0 ± 4.67 100 μm: 13.65 ± 3.61	Sa: 25 μm: 13.73 ± 1.75 50 μm: 14.45 ± 1.06 100 μm: 9.17 ± 0.48	Sa: 25 μm: 9.5 ± 3.50 50 μm: 11.47 ± 2.81 100 μm: 15.69 ± 4.89	Sa: 25 μm: 8.97 ± 3.39 50 μm: 8.41 ± 2.82 100 μm: 8.81 ± 3.51	NA	NA	NA	NA	
de Castro et al., 2023 [40]	Sa: (A) Cosmos Temp SLA: 0.088 ± 0.044 (B) Cosmos Temp DLP: 0.102 ± 0.034 (C) PriZma Bioprov: 0.066 ± 0.015 (D) Nanolab 3D: 0.201 ± 0.017	**-**	Sa: (A) Cosmos Temp SLA: 0.109 ± 0.031 (B) Cosmos Temp DLP: 0.104 ± 0.038 (C) PriZma Bioprov: 0.074 ± 0.014 (D) Nanolab 3D: 0.204 ± 0.012	**-**	Sa: (A) Cosmos Temp SLA: 0.110 ± 0.044 (B) Cosmos Temp DLP: 0.116 ± 0.047 (C) PriZma Bioprov: 0.067 ± 0.009 (D) Nanolab 3D: 0.202 ± 0.014	Sa: 0.050 ± 0.005	Toothbrushing wear: Sa: (A) Cosmos Temp SLA: 0.310 ± 0.041 (B) Cosmos Temp DLP: 0.284 ± 0.052 (C) PriZma Bioprov: 0.252.4 ± 0.064 (D) Nanolab 3D: 0.288 ± 0.015	Toothbrushing wear: Sa: (A) Cosmos Temp SLA: 0.329 ± 0.076 (B) Cosmos Temp DLP: 0.288 ± 0.043 (C) PriZma Bioprov: 0.268 ± 0.088 (D) Nanolab 3D: 0.281.9 ± 0.014	Toothbrushing wear: Sa: (A) Cosmos Temp SLA: 0.306 ± 0.039 (B) Cosmos Temp DLP: 0.308.9 ± 0.029 (C) PriZma Bioprov: 0.229.0 ± 0.049 (D) Nanolab 3D: 0.278.2 ± 0.013	Toothbrushing wear: Sa: 0.541.5 ± 0.656
Ortega NM et al., 2024 [51]	Ra: 2.23 ± 0.19	**-**	Ra: 3.19 ± 0.31	**-**	Ra: 1.20 ± 0.36	-	-	-	-	-
Alharbi et al., 2021 [53]	*Surface roughness prepolishing (Ra)* (a) 90: 9.423 ± 0.954; (b) 120: 2.474 ± 0.994; (c) 135: 1.926 ± 0.531; (d) 150: 2.523 ± 0.447; (e) 180: 0.787 ± 0.166					-	*Surface roughness after polishing (Ra)* (a) 90: 0.083 ± 0.032; (b) 120: 0.190 ± 0.108 (c) 135: 0.201 ± 0.056; (d) 150: 0.187 ± 0.132 (e) 180: 0.191 ± 0.099			-

NA: not applicable; Ra: arithmetic mean roughness; Sa: areal mean roughness

**Table 9:** Continuation of Surface Roughness (SR) Test Outcomes.

Author and Year	External Condition Inducing Change in SR	Testing Device	Authors’ Suggestions/Conclusions
Queiroz et al., 2024 [43]	NA	Contact profilometer (Rugosimeter model TR210, Time Group Inc.)	SR: 0° > 90° > 45°
Khanlar et al., 2023 [37]	NA	3D laser scanning confocal microscope (CLSM) (KEYENCE VK-X 150/160; KEYENCE	Surface roughness: significantly influenced by printing orientation SR: 45° > 90° > 0°
de Gois Moreira et al., 2025 [49]	NA	3D optical profilometer (Taylor Hobson-AMETEK)	Maximum SR displayed by e 30°/25 μm group At 50 μm printing layer thickness: SR: 45° > 60° > 30° > 0° > 90 °
de Castro et al., 2023 [40]	Toothbrushing	Laser confocal microscope (OLS5000, Olympus)	No influence of printing orientation on SR. SR: (A) Cosmos Temp SLA: 90° > 45° > 0° (B) Cosmos Temp DLP: 90° > 45° > 0° (C) PriZma Bioprov: 45° > 90° > 0° (D) Nanolab 3D: 45° > 90° > 0°
Ortega NM et al., 2024 [51]	-	Optical 3D measurement system (InfiniteFocusG5 plus)	Print orientation parameter significantly impacted the surface roughness. Ra: 45° > 0° > 90°
Alharbi et al., 2021 [53]	-	Contact stylus profilometer (Talysurf i60, Metek)	Surface roughness (Ra): Pre polishing: 90° > 150° > 120° > 135° > 180° After polishing: 135° > 180° > 120° > 150° > 90°

NA: not applicable; SR: Surface Roughness.

**Table 10:** Wear Resistance/Wear Volume Outcomes.

Author and Year	Lee et al., 2022 [45]	Wan et al., 2024 [46]
Mean volume loss (mm^3^)/RMS (µm) for conventional self-cure resin	Mean wear volume loss: 0.70 ± 0.15 RMS values: 11.88 ± 2.69	-
Mean volume loss (mm^3^)/RMS (µm) for specimens printed at 0°	Mean wear volume loss: 1.22 ± 0.63 RMS values: 12.14 ± 2.38	Mean wear volume loss (mm^3^): 50 μm: 1.208 ± 0.196; 100 μm: 1.010 ± 0.159
Mean volume loss (mm^3^)/RMS (µm) for specimens printed at 45°	Mean wear volume loss: 1.32 ± 0.48 RMS values: 13.78 ± 1.29	Mean wear volume loss (mm^3^): 50 μm: 0.886 ± 0.232; 100 μm: 0.854 ± 0.164
Mean volume loss (mm^3^)/RMS (µm) for specimens printed at 90°	Mean wear volume loss: 1.74 ± 0.41 RMS values: 16.46 ± 2.39	Mean wear volume loss (mm^3^): 50 μm: 1.063 ± 0.268; 100 μm: 1.136 ± 0.265
Duration of use/test	1 Week	60,000 cycles (equivalent to 3 months of clinical use)
Parameters of the chewing simulator	NA	Chewing simulator (vertical movement: 5 mm; horizontal movement: 2 mm) Vertical load: 5 KG; 0.8 Hz repetitive motion
Measuring device	Superimposition of scanned crowns	Superimposition of scanned specimens using a 3D metrology software (version 2018.1.2, 3D Systems).
Authors’ suggestions/conclusions	Wear volume loss and RMS: 90° > 45° > 0° > Conventional WR of 3D printed: 0° > 45° > 90° WR: Conv. Self-cure > 3D printed	Mean wear volume loss (mm^3^): 50 μm: 0° > 90° > 45°: 100 μm: 90° > 0° > 45°

NA: Not Applicable; RMS: root mean square; WR: wear resistance.

**Table 11:** Continuation of Flexural Strength (FS) Outcomes.

Author and Year	Mean Maximum Force at Fracture for Specimens Printed at 0° (in MPa)	Mean Maximum Force at Fracture for Specimens Printed at 30° (in MPa)	Mean Maximum Force at Fracture for Specimens Printed at 45° (in MPa)	Mean Maximum Force at Fracture for Specimens Printed at 60° (in MPa)	Mean Maximum Force at Fracture for Specimens Printed at 90° (in MPa)
Derban et al., 2021 [47]	Loading direction: Perpendicular NextDent: 117.24; Detax: 100.76	-	Loading direction: Perpendicular NextDent: 106.35; Detax: 85.05	-	Loading direction: Perpendicular NextDent: 117.84; Detax: 113.98
Queiroz et al., 2024 [43]	236.20 ± 29.73	-	155.80 ± 36.19	-	138.70 ± 48.20
Mudhaffer et al., 2025 [38]	(A) ND: *(1) DW:* (i) 24 h: 90.5 ± 4.1; (ii) 1 m: 81.8 ± 3.6; (iii) 3 m: 87.6 ± 4.8 *(2) AS:* (i) 24 h: 87.7 ± 2.7 (ii) 1 m: 72.9 ± 1.4; (iii) 3 m: 81.2 ± 3.5 (B) DT: *(1) DW:* (i) 24 h: 79.6 ± 3.0 (ii) 1 m: 78.3 ± 8.6; (iii) 3 m: 79.4 ± 5.7 *(2) AS:* (i) 24 h: 80.4 ± 2.2 (ii) 1 m: 79.5 ± 2.2; (iii) 3 m: 78.1 ± 7.0 (C) GC: *(1) DW:* (i) 24 h: 69.4 ± 3.5 (ii) 1 m: 82.6 ± 3.3; (iii) 3 m: 88.8 ± 7.2 *(2) AS:* (i) 24 h: 74.4 ± 4.0 (ii) 1 m: 78.7 ± 8.3; (iii) 3 m: 87.4 ± 8.6	-	(A) ND: *(1) DW:* (i) 24 h: 102.6 ± 5.6 (ii) 1 m: 83.6 ± 1.5; (iii) 3 m: 89.6 ± 1.7 *(2) AS:* (i) 24 h: 94.3 ± 2.1 (ii) 1 m: 77.5 ± 1.2; (iii) 3 m: 86.6 ± 3.81 (B) DT: *(1) DW:* (i) 24 h: 81.7 ± 2.4 (ii) 1 m: 87.5 ± 10.0; (iii) 3 m: 92.1 ± 3.8 *(2) AS:* (i) 24 h: 80.0 ± 4.2 (ii) 1 m: 79.2 ± 3.2; (iii) 3 m: 87.7 ± 3.2 (C) GC: *(1) DW:* (i) 24 h: 74.9 ± 3.3 (ii) 1 m: 76.8 ± 4.9: (iii) 3 m: 79.7 ± 5.6 *(2) AS:* (i) 24 h: 85.4 ± 5.5 (ii) 1 m: 75.4 ± 3.3: (iii) 3 m: 76.9 ± 9.5	-	(A) ND: *(1) DW:* (i) 24 h: 106.2 ± 4.9 (ii) 1 m: 83.2 ± 1.5; (iii) 3 m: 89.9 ± 1.9 *(2) AS:* (i) 24 h: 95.5 ± 3.2 (ii) 1 m: 79.9 ± 1.4; (iii) 3 m: 91.3 ± 2.1 (B) DT: *(1) DW:* (i) 24 h: 87.7 ± 2.1 (ii) 1 m: 102.0 ± 1.2; (iii) 3 m: 93.4 ± 6.9 *(2) AS:* (i) 24 h: 86.8 ± 1.1 (ii) 1 m: 86.6 ± 3.4; (iii) 3 m: 93.3 ± 1.7 (C) GC: *(1) DW:* (i) 24 h: 82.4 ± 3.4 (ii) 1 m: 90.3 ± 3.8; (iii) 3 m: 89.7 ± 5.8 *(2) AS:* (i) 24 h: 85.2 ± 3.8 (ii) 1 m: 82.9 ± 4.3; (iii) 3 m: 89.2 ± 6.9
Casucci et al., 2024 [48]	(A) VS: 132.52 ± 35.25 (B) VP: 156.56 ± 25.58 (C) BT: 113.37 ± 31.93 (D) SC: 110.41 ± 36.13	-	(A) VS: 133.04 ± 22.12 (B) VP: 130.46 ± 12.33 (C) BT: 148.91 ± 21.23 (D) SC: 136.33 ± 15.38	-	(A) VS: 134.24 ± 14.94 (B) VP: 116.97 ± 34.87 (C) BT: 100.96 ± 16.66 (D) SC: 114.85 ± 17.72
de Gois Moreira et al., 2025 [49]	Without thermocycling 25 μm: 48.6 ± 6.6; 50 μm: 41.0 ± 6.4 100 μm: 39.4 ± 5.4 With thermocycling 25 μm: 45.8 ± 3.1; 50 μm: 34.3 ± 5.8 100 μm: 39.0 ± 4.4	Without thermocycling 25 μm: 51.0 ± 4.5; 50 μm: 44.0 ± 2.3 100 μm: 34.8 ± 4.5 With thermocycling 25 μm: 54.5 ± 6.4; 50 μm: 40.5 ± 38 100 μm: 36.0 ± 5.0	Without thermocycling 25 μm: 41.0 ± 3.9; 50 μm: 43.3 ± 2.4 100 μm: 43.4 ± 4.0 With thermocycling 25 μm: 57.7 ± 3.1; 50 μm: 41.3 ± 4.7 100 μm: 33.6 ± 4.6 N	Without thermocycling 25 μm: 50.3 ± 3.0; 50 μm: 42.8 ± 4.6 100 μm: 38.7 ± 3.1 With thermocycling 25 μm: 47.6 ± 4.8; 50 μm: 37.9 ± 3.0 100 μm: 37.1 ± 3.4	Without thermocycling 25 μm: 46.2 ± 4.9; 50 μm: 47.4 ± 3.5 100 μm: 37.7 ± 3.4 With thermocycling 25 μm: 63.0 ± 4.5; 50 μm: 53.6 ± 5.1 100 μm: 47.7 ± 3.7
de Castro et al., 2022 [25]	Without aging Cosmos Temp-SLA: 31.3 ± 3.6 Cosmos Temp-DLP: 72.3 ± 5.8 PriZma BioProv: 86.2 ± 4.1 Nanolab 3D: 66.6 ± 9.2 After 1-year aging Cosmos Temp-SLA: 71.0 ± 10.1 Cosmos Temp-DLP: 41.3 ± 4.9 PriZma BioProv: 89.3 ± 3.4 Nanolab 3D: 41.2 ± 1.6	-	Without aging Cosmos Temp-SLA: 35.4 ± 5 Cosmos Temp-DLP: 60.5 ± 9.8 PriZma BioProv: 84.8 ± 5.8 Nanolab 3D: 70.8 ± 3.0 After 1-year aging Cosmos Temp-SLA: 90.7 ± 12.2 Cosmos Temp-DLP: 40.9 ± 6.1 PriZma BioProv: 91.8 ± 6.6 Nanolab 3D: 39.7 ± 10.3	-	Without aging Cosmos Temp-SLA: 39.2 ± 2.8 Cosmos Temp-DLP: 74.5 ± 3.7 PriZma BioProv: 80.3 ± 4.4 Nanolab 3D: 63.0 ± 5.5 After 1-year aging Cosmos Temp-SLA: 109.3 ± 13.9 Cosmos Temp-DLP: 35.6 ± 3.7 PriZma BioProv: 86.1 ± 8.1 Nanolab 3D: 39.4 ± 4.1
Espinar et al., 2024 [41]	(A) Detax Freeprint Temp: 98.81 ± 13.54 (B) Formlabs Temporary CB: 127.83 ± 20.35 (C) Formlabs Permanent Crown: 142.62 ± 12.98 (D) GCT-GC: 94.28 ± 11.03	-	-	-	(A) Detax Freeprint Temp: 111.36 ± 14.80 (B) Formlabs Temporary CB: 129.29 ± 23.02 (C) Formlabs Permanent Crown: 120.73 ± 18.07 (D) GCT-GC: 91.68 ± 11.78
Kaiahara et al., 2025 [50]	80.90 ± 4.0	-	90.10 ± 8.45	-	114.71 ± 7.61

ND: Nextdent CB MFH; DT: Dima CB temp; GC: GC temp print; DW: Distilled water; AS: artificial saliva; h: hours; m = month; VS: Varseo smile teeth; VP: V-print C&B temp; BT: Bego Triniq; SC: Sprintray Crown.

**Table 12:** Flexural Strength (FS) Outcomes.

Author and Year	Mean/Median of Maximum Force at Fracture for Specimens Fabricated by Milling/Conventional Technique (in MPa)	Exposure Agent/Aging Method	Testing Machine	Authors’ Recommendations/ Conclusions
Derban et al., 2021 [47]	-	NM	Universal testing machine	FS: For both NextDent and Detax: 90° > 0° > 45° NextDent > Detax
Queiroz et al., 2024 [43]	-	No	Universal testing machine	FS: 0° > 45° > 90°
Mudhaffer et al., 2025 [38]	-	DW and AS (24 h, 1 m, 3 m)	Universal testing machine	FS is significantly influenced by printing orientation. FS: 90° > 45° > 0° Effect of aging is minimal and varies with each material. All materials met the minimum FS requirement of 80 MPa when printed at 90°.
Casucci et al., 2024 [48]	-	DW (24 h)	Universal testing machine	FS is significantly influenced by printing orientation, with different materials displaying varied results. FS: (A) VS: 90° > 45° > 0° (B) VP: 0° > 45° > 90° (C) BT: 45° > 0° > 90° (D) SC: 45° > 90° > 0°
de Gois Moreira et al., 2025 [49]	-	Thermocycling (10,000 cycles)	Universal testing machine	FS is best for specimens printed at a print layer thickness of 25 μm combined with build angles of 90° and 45°.
de Castro et al., 2022 [25]	Vita Temp (Control) (Milled): 94.8 ± 3.3 After 1-year aging: 83.7 ± 8.2	1 year water storage	Universal testing machine	After 1-year water storage, Cosmos-SLA printed at 90° showed the highest FS. FS varied with material and printing orientation. FS After 1-year aging Cosmos Temp-SLA: 90° > 45° > 0° Cosmos Temp-DLP: 0° > 45° > 90° PriZma BioProv: 45° > 0° > 90° Nanolab 3D: 0° > 45° > 90°
Espinar et al., 2024 [41]	-	-	Universal testing machine	Printing orientation did not influence flexural strength. (A) Detax Freeprint Temp: 90° > 0° (B) Formlabs Temporary CB: 90° > 0° (C) GCT-GC: 0° > 90°
Kaiahara et al., 2025 [50]	78.13 ± 7.94	-	Universal testing machine	Printing orientation significantly influences flexural strength. FS: 90° > 45° > 0° FS: 3D printed > Milled

DW: distilled water; AS: artificial saliva; h: hours; m = month; NM: Not Mentioned.

**Table 13:** Elastic Modulus Outcomes.

Author and Year	Espinar et al., 2024 [41]
Mean Elastic Modulus of specimens printed at 0° (MPa)	(A) Detax Freeprint Temp: 2552.55 ± 155.84 (B) Formlabs Temporary CB: 4426.70 ± 512.76 (C) Formlabs Permanent Crown: 4262 ± 442.49 (D) GCT-GC: 2898.73 ± 267.65F
Mean Elastic Modulus of specimens printed at 90° (MPa)	(A) Detax Freeprint Temp: 2750.00 ± 140.31 (B) Formlabs Temporary CB: 6639.50 ± 231.74 (C) Formlabs Permanent Crown: 4349.20 ± 230.93 (D) GCT-GC: 2887.67 ± 176.63
Testing Machine	Universal testing machine
Authors’ Recommendations/Conclusions	Printing orientation did not influence elastic modulus. (A) Detax Freeprint Temp: 90° > 0° (B) Formlabs Temporary CB: 90° > 0° (C) GCT-GC: 0° > 90°

**Table 14:** Assessment of strength of evidence using GRADE approach.

Outcome	Effect of Different Printing Orientations on the Mechanical Properties of 3D-Printed Provisional Fixed Dental Prosthesis [25,34,39,41,42,43,45,46,47,48,49,50,51,53]	Effect of Different Printing Orientations on the Physical Properties of 3D-Printed Provisional Fixed Dental Prosthesis [33,40,44]
Inconsistency	NP	NP
Indirectness	NP	NP
Imprecision	NP	NP
Risk of Bias	P	P
Publication Bias	NP	NP
Strength of Evidence	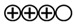	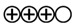

NP: not present; P: present.

## Data Availability

The data that support the findings of this study are available from the corresponding author upon reasonable request.

## Supplementary Materials

The following supporting information can be downloaded at: https://www.mdpi.com/article/10.3390/jfb16080278/s1, Table S1: Search terms and strategy for the electronic databases; Table S2: Quality analyses results of the included studies.
