# Supplementary material for: The Influence of Printing Orientation on the Properties of 3D-Printed Polymeric Provisional Dental Restorations: A Systematic Review and Meta-Analysis
Source: J Funct Biomater. 2025 Jul 31;16(8):278. doi: 10.3390/jfb16080278 (PMC12387784; doi:10.3390/jfb16080278)
Supplement: Supplementary file 1 [file jfb-16-00278-s001.zip › Supplementary file 1.pdf]

Supplementary Table S1. Search terms and strategy for the electronic databases.

Database	Combination of Search Terms and Strategy	Number of Titles
MEDLINE/PubMed	(("denture, partial, fixed"[MeSH Terms] OR "dental restoration, temporary"[MeSH Terms] OR "Dental Prosthesis"[MeSH Terms] OR "crowns"[MeSH Terms] OR "denture, partial, temporary"[MeSH Terms] OR "Tooth Crown"[MeSH Terms] OR "Stereolithography"[MeSH Terms] OR "printing, three dimensional"[MeSH Terms] OR "Computer-Aided Design"[MeSH Terms] OR "provisional crown"[Title/Abstract] OR "provisional fixed partial denture"[Title/Abstract] OR "Provisional Fixed Dental Prosthesis"[All Fields] OR "Temporary Crown and Bridge"[Title/Abstract] OR "temporary crown"[Title/Abstract] OR "Temporary dental restoration"[Title/Abstract] OR "interim restoration"[Title/Abstract] OR "interim crown"[Title/Abstract] OR "interim resin"[Title/Abstract] OR "interim fixed partial denture"[Title/Abstract] OR "Provisional Dental Restoration"[Title/Abstract] OR "provisional resin"[Title/Abstract] OR "3d print*"[Title/Abstract] OR "Rapid prototyping"[Title/Abstract] OR "additive manufactur*"[Title/Abstract] OR "computer-aided manufacturing"[Title/Abstract] OR "Computer-Assisted Designing"[Title/Abstract] OR "Computer-Assisted manufacturing"[Title/Abstract] OR "cad cam"[Title/Abstract] OR "Three-Dimensional Printing"[Title/Abstract] OR "Digital light processing"[All Fields] OR "3D-printed resin"[All Fields]) AND (("all"[Filter] NOT "preprint"[Publication Type]) AND "humans"[MeSH Terms] AND "english"[Language]) AND (("printing orientation*"[Title/Abstract] OR "Build Orientation"[Title/Abstract] OR "build up angle*"[Title/Abstract] OR "Printing Direction"[Title/Abstract] OR "3d printing parameter*"[Title/Abstract] OR "Orientation"[Title/Abstract] OR "Printing Angle"[Title/Abstract]) AND (("all"[Filter] NOT "preprint"[Publication Type]) AND "humans"[MeSH Terms] AND "english"[Language])) AND (("Mechanical Phenomena"[MeSH Terms] OR "Physical Phenomena"[MeSH Terms] OR "Hardness Tests"[MeSH Terms] OR "Flexural Strength"[MeSH Terms] OR "elasticity"[MeSH Terms] OR "elastic modulus"[MeSH Terms] OR "compressive strength"[MeSH Terms] OR "Tensile Strength"[MeSH Terms] OR "Shear strength"[MeSH Terms] OR "hardness"[MeSH Terms] OR "Mechanical Tests"[MeSH Terms] OR "Dental Restoration Wear"[MeSH Terms] OR "solubility"[MeSH Terms] OR "viscosity"[MeSH Terms] OR "Optical Phenomena"[MeSH Terms] OR "color"[MeSH Terms] OR "Color Stability"[Title/Abstract] OR "Translucency"[Title/Abstract] OR "Color Change"[Title/Abstract] OR ("tarnish"[All Fields] OR "Brittleness"[Title/Abstract] OR "Toughness"[Title/Abstract] OR "Flexibility"[Title/Abstract] OR "Mechanical properties"[Title/Abstract] OR "Physical properties"[Title/Abstract] OR "fracture toughness"[Title/Abstract] OR "fracture load"[Title/Abstract] OR "Fracture resistance"[Title/Abstract] OR "fracture strength"[Title/Abstract] OR "Yield strength"[Title/Abstract] OR "Fatigue strength"[Title/Abstract] OR "fatigue test"[Title/Abstract] OR "surface roughness"[Title/Abstract] OR "water absorption"[Title/Abstract] OR "Microhardness"[Title/Abstract] OR "wear resistance"[Title/Abstract] OR "surface wear"[Title/Abstract] OR "tarnishes"[All Fields] OR "Shear Bond Strength"[Title/Abstract] OR "Elastic strength"[Title/Abstract] OR "Abrasion"[Title/Abstract] OR "Abrasion resistance"[Title/Abstract] OR "tarnishing"[All Fields]) OR "corrosion"[Title/Abstract] OR "Creep"[Title/Abstract] OR "compression test"[Title/Abstract] OR "Martens hardness"[Title/Abstract] OR "Indentation modulus"[Title/Abstract]) AND "english"[Language]))	222
Scopus	(TITLE-ABS-KEY ("fixed partial denture" OR "temporary dental restoration" OR "Dental Prosthesis" OR crowns OR "temporary partial denture " OR "Tooth Crown" OR "Stereolithography" OR "three dimensional printing" OR "Computer-Aided Design" OR "provisional crown" OR "provisional fixed partial denture" OR "Provisional Dental Restoration" OR "Provisional Fixed Dental Prosthesis" OR "Temporary Crown and Bridge" OR "temporary crown" OR "Temporary dental restoration" OR "interim restoration" OR "interim crown" OR "interim resin" OR "interim fixed partial denture" OR "provisional resin" OR "3d printing" OR "Rapid prototyping" OR "additive manufacturing" OR "computer-aided manufacturing" OR "Computer-Assisted Designing" OR "Computer-Assisted manufacturing" OR "cad cam" OR "Three-Dimensional Printing" OR "Digital light processing" OR "3D-printed resin") AND TITLE-ABS-KEY ("printing orientation" OR "Build Orientation" OR "buildup angle" OR "Printing Direction" OR "3d printing parameter" OR "Orientation" OR printing AND angle) AND TITLE-ABS-KEY ("Flexural Strength" OR elasticity OR "elastic modulus" OR "compressive strength" OR "Tensile Strength" OR "Shear strength" OR hardness OR "Dental Restoration Wear" OR solubility OR "Optical Phenomena" OR color OR "Color Stability" OR translucency OR brittleness OR toughness OR flexibility OR "Mechanical properties" OR "Physical properties" OR "fracture toughness" OR "fracture load" OR "Fracture resistance" OR "fracture strength" OR "Yield strength" OR "Fatigue strength" OR "surface roughness" OR "water absorption" OR microhardness OR "wear resistance" OR tarnish OR "Abrasion resistance" OR corrosion OR creep OR "compression test" OR "Martens hardness" OR "Indentation modulus"	135

	)) AND (LIMIT-TO (SUBJAREA , "MEDI") OR LIMIT-TO (SUBJAREA , "DENT")) AND (LIMIT-TO (DOCTYPE , "ar")) AND (LIMIT-TO (LANGUAGE , "English"))	
Web of Sciences (Core collection)	#1 (P)<br>(TS = ("fixed partial denture" OR "temporary dental restoration" OR "Dental Prosthesis" OR crowns OR "temporary partial denture " OR "Tooth Crown" OR "Stereolithography" OR "three dimensional printing" OR "Computer-Aided Design" OR "provisional crown" OR "provisional fixed partial denture" OR "Provisional Dental Restoration" OR "Provisional Fixed Dental Prosthesis" OR "Temporary Crown and Bridge" OR "temporary crown" OR "Temporary dental restoration" OR "interim restoration" OR "interim crown" OR "interim resin" OR "interim fixed partial denture" OR "provisional resin" OR "3d printing" OR "Rapid prototyping" OR "additive manufacturing" OR "computer-aided manufacturing" OR "Computer-Assisted Designing" OR "Computer-Assisted manufacturing" OR "cad cam" OR "Three-Dimensional Printing" OR "Digital light processing" OR "3D-printed resin")) AND LANGUAGE: (English)<br>Indexes=SCI-EXPANDED, SSCI, A&HCI, CPCI-S, CPCI-SSH, ESCI, CCR-EXPANDED, IC Timespan = All years<br>#2 (I & C)<br>(TS = ("printing orientation" OR "Build Orientation" OR "buildup angle" OR "Printing Direction" OR "3d printing parameter" OR "Orientation" OR printing AND angle)) AND LANGUAGE: (English) Indexes=SCI-EXPANDED, SSCI, A&HCI, CPCI-S, CPCI-SSH, ESCI, CCR-EXPANDED, IC Timespan = All years<br>#3 (O)<br>(TS=("Flexural Strength" OR elasticity OR "elastic modulus" OR "compressive strength" OR "Tensile Strength" OR "Shear strength" OR hardness OR "Dental Restoration Wear" OR solubility OR "Optical Phenomena" OR color OR "Color Stability" OR translucency OR brittleness OR toughness OR flexibility OR "Mechanical properties" OR "Physical properties" OR "fracture toughness" OR "fracture load" OR "Fracture resistance" OR "fracture strength" OR "Yield strength" OR "Fatigue strength" OR "surface roughness" OR "water absorption" OR microhardness OR "wear resistance" OR tarnish OR "Abrasion resistance" OR corrosion OR creep OR "compression test" OR "Martens hardness" OR "Indentation modulus"))AND LANGUAGE: (English)<br>Indexes = SCI-EXPANDED, SSCI, A&HCI, CPCI-S, CPCI-SSH, ESCI, CCR-EXPANDED, IC Timespan = All years<br>#3 AND #2 AND #1<br>Indexes = SCI-EXPANDED, SSCI, A&HCI, CPCI-S, CPCI-SSH, ESCI, CCR-EXPANDED, IC Timespan = All years and English (Languages)	103
Cochrane Library	ID Search Hits<br>#1 MeSH descriptor: [Denture, Partial, Fixed] explode all trees;<br>#2 MeSH descriptor: [Dental Restoration, Temporary] explode all trees<br>#3 MeSH descriptor: [Dental Prosthesis] explode all trees<br>#4 MeSH descriptor: [Crowns] explode all trees<br>#5 MeSH descriptor: [Denture, Partial, Temporary] explode all trees<br>#6 MeSH descriptor: [Tooth Crown] explode all trees<br>#7 MeSH descriptor: [Stereolithography] explode all trees<br>#8 MeSH descriptor: [Printing, Three-Dimensional] explode all trees<br>#9 MeSH descriptor: [Computer-Aided Design] explode all trees<br>#10 provisional fixed partial denture<br>#11 provisional crown<br>#12 Provisional Fixed Dental Prosthesis<br>#13 Temporary Crown and Bridge<br>#14 temporary crown<br>#15 Temporary dental restoration<br>#16 interim restoration<br>#17 interim crown<br>#18 interim resin<br>#19 interim fixed partial denture<br>#20 Provisional Dental Restoration<br>#21 provisional resin<br>#22 3d printing<br>#23 Rapid prototyping<br>#24 additive manufacturing<br>#25 computer-aided manufacturing<br>#26 Computer-Assisted Designing	34

	#27 Computer-Assisted manufacturing<br>#28 cad cam<br>#29 Three-Dimensional Printing<br>#30 Digital light processing<br>#31 3D-printed resin<br>#32 printing orientation<br>#33 Build Orientation<br>#34 buildup angle<br>#35 Printing Direction<br>#36 3d printing parameter<br>#37 Orientation<br>#38 printing angle<br>#39 Flexural Strength<br>#40 elasticity<br>#41 elastic modulus<br>#42 compressive strength<br>#43 Tensile Strength<br>#44 Shear strength<br>#45 hardness<br>#46 Dental Restoration Wear<br>#47 solubility<br>#48 Optical Phenomena<br>#49 color<br>#50 Color Stability<br>#51 translucency<br>#52 brittleness<br>#53 toughness<br>#54 flexibility<br>#55 Mechanical properties<br>#56 Physical properties<br>#57 fracture toughness<br>#58 fracture load<br>#59 Fracture resistance<br>#60 fracture strength<br>#61 Yield strength<br>#62 Fatigue strength<br>#63 surface roughness<br>#64 water absorption<br>#65 microhardness<br>#66 wear resistance<br>#67 tarnish<br>#68 Abrasion resistance<br>#69 corrosion<br>#70 creep<br>#71 compression test<br>#72 Martens hardness<br>#73 Indentation modulus<br>#74 #1 OR #2 OR #3 OR #4 OR #5 OR #6 OR #7 OR #8 OR #9 OR #10 OR #11 OR #12 OR #13 OR #14 OR #15 OR #16 OR #17 OR #18 OR #19 OR #20 OR #21 OR #22 OR #23 OR #24 OR #25 OR #26 OR #27 OR #28 OR #29 OR #30 OR #31<br>#75 #32 OR #33 OR #34 OR #35 OR #36 OR #37 OR #38<br>#76 #39 OR #40 OR #41 OR #42 OR #43 OR #44 OR #45 OR #46 OR #47 OR #48 OR #49 OR #50 OR #51 OR #52 OR #53 OR #54 OR #55 OR #56 OR #57 OR #58 OR #59 OR #60 OR #61 OR #62 OR #63 OR #64 OR #65 OR #66 OR #67 OR #68 OR #69 OR #70 OR #71 OR #72 OR #73<br>#77 #74 AND #75 AND #76	

*: Truncation, P: Population, I: Intervention, C: Comparator, O: Outcome.

Supplementary Table S2: Quality analyses results of the included studies

Item → Studies ↓	1	2a	2b	3	4	5	6	7	8	9	10	11	12	13	14
Alharbi et al 2016 [34]	Y	Y	Y	Y	Y	Y	N	N	N	N	Y	Y	Y	Y	N
Derban et al, 2021 [47]	Y	Y	Y	Y	Y	Y	N	N	N	N	Y	Y	N	Y	Y
Alharbi et al, 2021 [53]	Y	Y	Y	Y	Y	Y	N	N	N	Y	Y	Y	Y	Y	N
Lee at al, 2022, Korea [45]	Y	Y	Y	Y	Y	Y	N	N	N	N	Y	Y	Y	Y	Y
de Castro et al, 2022 [25]	Y	Y	Y	Y	Y	Y	N	N	N	N	Y	Y	Y	Y	N
Lee et al, 2022 [33]	Y	Y	Y	Y	Y	Y	N	N	N	N	Y	Y	Y	Y	N
de Castro et al, 2023 [40]	Y	Y	Y	Y	Y	Y	N	N	N	N	Y	Y	Y	Y	N
Farkas et al, 2023 [35]	Y	Y	Y	Y	Y	Y	N	N	N	N	Y	Y	Y	Y	Y
Alkhateeb et al, 2023 [42]	Y	Y	Y	Y	Y	Y	N	N	N	N	Y	Y	Y	Y	Y
Espinar et al, 2023 [44]	Y	Y	Y	Y	Y	Y	N	N	N	N	Y	Y	Y	Y	Y
Espinar et al, 2024 [41]	Y	Y	Y	Y	Y	Y	N	N	N	N	Y	Y	Y	Y	Y
Queiroz et al, 2024 [43]	Y	Y	Y	Y	Y	Y	N	N	N	N	Y	Y	Y	Y	Y
Mudhaffer et al, 2024 [38]	Y	Y	Y	Y	Y	Y	N	N	N	N	Y	Y	Y	Y	N
Casucci et al, 2024 [48]	Y	Y	Y	Y	Y	Y	N	N	N	N	Y	Y	Y	Y	Y
Wan et al, 2024 [46]	Y	Y	Y	Y	Y	Y	N	N	N	N	Y	Y	Y	Y	N
Ortega NM et al, 2024 [51]	Y	Y	Y	Y	Y	Y	N	N	N	N	Y	Y	Y	N	N
Aljehani et al, 2024 [36]	Y	Y	Y	Y	Y	Y	N	N	N	N	Y	Y	Y	N	Y
Khanlar et al, 2024 [37]	Y	Y	Y	Y	Y	Y	N	N	N	N	Y	Y	Y	Y	N
Mudhaffer et al, 2024 [39]	Y	Y	Y	Y	Y	Y	N	N	N	N	Y	Y	Y	Y	N
de Gois Moreira et al, 2025 [49]	Y	Y	Y	Y	Y	Y	N	N	N	N	Y	Y	Y	Y	Y
Kaiahara et al , 2025 [50]	Y	Y	Y	Y	Y	Y	N	N	N	N	Y	Y	Y	Y	Y
