# Supplementary material for: The Influence of Printing Orientation on the Properties of 3D-Printed Polymeric Provisional Dental Restorations: A Systematic Review and Meta-Analysis
Source: J Funct Biomater. 2025 Jul 31;16(8):278. doi: 10.3390/jfb16080278 (PMC12387784; doi:10.3390/jfb16080278)
Supplement: Supplementary file 1 [file jfb-16-00278-s001.zip › Supplementary file 2 PRISMA 2020 abstract checklist.pdf]

PRISMA 2020 for Abstracts Checklist

Section and Topic	Item #	Checklist item	Reported (Yes/No)
**TITLE**			
Title	1	Identify the report as a systematic review.	Yes (Page 1)
**BACKGROUND**			
Objectives	2	Provide an explicit statement of the main objective(s) or question(s) the review addresses.	Yes (Page 1)
**METHODS**			
Eligibility criteria	3	Specify the inclusion and exclusion criteria for the review.	Yes (Page 1)
Information sources	4	Specify the information sources (e.g. databases, registers) used to identify studies and the date when each was last searched.	Yes (Page 1)
Risk of bias	5	Specify the methods used to assess risk of bias in the included studies.	Yes (Page 1)
Synthesis of results	6	Specify the methods used to present and synthesise results.	Yes (Page 1)
**RESULTS**			
Included studies	7	Give the total number of included studies and participants and summarise relevant characteristics of studies.	Yes (Page 1)
Synthesis of results	8	Present results for main outcomes, preferably indicating the number of included studies and participants for each. If meta-analysis was done, report the summary estimate and confidence/credible interval. If comparing groups, indicate the direction of the effect (i.e. which group is favoured).	Yes (Page 1)
**DISCUSSION**			
Limitations of evidence	9	Provide a brief summary of the limitations of the evidence included in the review (e.g. study risk of bias, inconsistency and imprecision).	Yes (Page 1)
Interpretation	10	Provide a general interpretation of the results and important implications.	Yes (Page 1)
**OTHER**			
Funding	11	Specify the primary source of funding for the review.	No
Registration	12	Provide the register name and registration number.	Yes
